# Multidisciplinary Evaluation of a 10‐Year Restoration Program for Two Endangered Atlantic Salmon ( *Salmo salar* ) Populations

**DOI:** 10.1111/eva.70289

**Published:** 2026-06-29

**Authors:** Louarn Fauchet, Anne‐Laure Ferchaud, Jean‐Christophe Guay, Jean‐Sébastien Moore, Nicolas Derôme, Julien April, Louis Bernatchez

**Affiliations:** ^1^ Département de Biologie Université Laval Québec City Québec Canada; ^2^ Institut de Biologie intégrative et Des systèmes (IBIS) Université Laval Québec City Québec Canada; ^3^ Ressources Aquatiques Québec Québec City Québec Canada; ^4^ Parks Canada Office of the Chief Ecosystem Scientist, Protected Areas Establishment and Conservation Directorate Québec City Québec Canada; ^5^ Direction Environnement, Expertise Milieu Naturel Hydro‐Québec Québec City Québec Canada; ^6^ Direction Principale de L'expertise Sur la Faune Aquatique, ministère de L'environnement, de la Lutte Contre les Changements Climatiques, de la Faune et des Parcs Québec City Québec Canada

## Abstract

Supportive breeding programs are widely implemented to counteract demographic collapse in threatened populations. Their long‐term success, however, depends on maintaining genetic diversity while ensuring that released individuals contribute effectively to wild populations. In Atlantic salmon ( *Salmo salar* ), uncertainties remain regarding the capacity of captive broodstocks to preserve genetic variation and the demographic and genetic consequences of supplementation. Here, we evaluate the outcomes of a decade‐long restoration program targeting two genetically differentiated but geographically proximate Atlantic salmon populations in the Romaine watershed (Romaine and Puyjalon population, Québec, Canada). Using microsatellite data, we performed population assignment, parentage analyses, and temporal estimates of effective number of breeders and genetic diversity. We assessed broodstock performance, the contribution of stocked individuals to wild populations, and the impact of two egg incubation treatments on fry to smolt survival. Despite sustained efforts to maintain large and representative broodstocks, the failure of wild‐caught juvenile salmon to reach maturity substantially reduced the number of breeders, representing the greatest loss of genetic potential in the hatchery broodstock. Nevertheless, partial factorial mating and kinship‐based management allowed us to avoid inbreeding and preserved levels of heterozygosity and allelic richness comparable to those observed in wild populations. Stocked individuals contributed around 25% to juvenile population size in both populations, without reducing effective population size or eroding genetic differentiation. Estimates of total effective population size consistently exceeded those of wild components alone, indicating no evidence of a Ryman–Laikre effect. Finally, egg incubation in the water of the Romaine River increased survival to smolt stage for genetically Romaine individuals but not for individuals from Puyjalon, suggesting local adaptation to early rearing conditions. These results demonstrate that, when carefully designed and genetically monitored, supportive breeding can reinforce depleted salmon populations without compromising genetic diversity and integrity.

## 1 Introduction

Over the past five decades, aquatic biodiversity has declined at an unprecedented rate and current efforts may not be sufficient to avert future biodiversity loss (Dias et al. 2017; Dudgeon and Strayer 2024; Logez et al. 2025; WWF 2024). Marine and freshwater species are increasingly threatened by aquatic territory use (Jaureguiberry et al. 2022), climate change (Nagelkerken et al. 2023), overexploitation by industrial (Du et al. 2021) and recreational (Abbott et al. 2022) fisheries, water pollution (Jan et al. 2022), invasive species (Bailey et al. 2020), and habitat degradation (Williams‐Subiza and Epele 2021). A recent global assessment revealed that one quarter of freshwater fauna is threatened with extinction (Sayer et al. 2025), and in Canada alone, nearly 12% of freshwater species are at risk (Desforges et al. 2022). In response to these alarming declines, numerous conservation interventions have been developed to restore depleted populations.

Supportive breeding—the artificial breeding and release of individuals into the wild to supplement natural reproduction—has become a widely used tool for restoring depleted or extirpated populations (Araki and Schmid 2010; Casimiro et al. 2022; Lipscomb et al. 2025). The primary goal is to provide demographic reinforcement while minimizing genetic alteration, ensuring that released individuals can successfully reproduce and sustain viable populations without a long‐term dependence on hatchery supplementation (Araki et al. 2008; Fraser 2008). Supportive breeding programs have been implemented across a wide range of aquatic taxa like amphibians (Karlsdóttir et al. 2021; Tapley et al. 2015), invertebrates (Carranza and Zu Ermgassen 2020; Geist et al. 2023; Grant et al. 2017; Li and Jiao 2015), as well as marine (MacNamara et al. 2022), freshwater (Buckley et al. 2024; Stoeckle et al. 2022; Tave 2025), and anadromous fishes (Janowitz‐Koch et al. 2019; Patrick et al. 2006). As a result of their substantial cultural and economic value as well as their severe population declines, anadromous salmonids are among the most frequent targets of supportive breeding programs (Dadswell et al. 2022; Gardner 2011; Williams et al. 2024). Indeed, species that depend on connectivity between marine and freshwater environments face disproportionate risks (Waldman and Quinn 2022) particularly through hydrological alteration resulting from dam construction, which fragments habitats and disrupts natural flow regimes (Chan et al. 2025; Huang and Li 2024). These constraints have made supportive breeding a central management tool for salmonid conservation. However, McMillan et al. (2023) reported that only 29% of studies described beneficial effects of supportive breeding on wild salmon populations, while 30% documented adverse effects. This variability in reported outcomes highlights the challenges associated with implementing effective restoration programs and the need to develop international guidelines (Grant et al. 2017; Lennox et al. 2021).

The long‐term success of such programs depends on minimizing unintended genetic and ecological consequences. Salmonids are well known to exhibit strong local adaptation, largely driven by fine‐scale homing behavior (Fraser et al. 2011; May et al. 2023; Taylor 1991). Hence, the use of locally adapted wild broodstock is a key requirement in supportive breeding programs, as non‐native fish have consistently shown lower reproductive success than their local counterparts (Egal et al. 2025; McGinnity et al. 2004; Peterson et al. 2014). At the population scale, the use of non‐local fish has been empirically shown to alter population genetic structure and erode local adaptation (Ayllon et al. 2006; Grandjean et al. 2009; Perrier et al. 2013), which is a critical component for facing future environmental change.

The loss of genetic diversity, which can occur through unequal parental contributions or the repeated use of a limited number of breeders, may compromise short‐ and long‐term population recovery (Allendorf and Phelps 1980; Bouchard et al. 2022; Christie, Marine, French, Waples, and Blouin 2012). Reduced allelic diversity increases the risk of inbreeding depression, leading to the accumulation of deleterious alleles that can reduce survival or reproductive success (Christie et al. 2014; Duchesne and Bernatchez 2002; Favé et al. 2008). Therefore, maximizing genetic diversity within the broodstock is essential and can be achieved by increasing the number of breeders, maintaining balanced sex ratios, and implementing partial factorial mating designs, which together increase the effective size (*N*_*e*_) of the broodstock (Fisch et al. 2015; Gilbey 2022; Waples 2024). Broodstock *N*_*e*_ is a key factor in supportive breeding programs as it reflects the number of individuals that contribute genes to the next generation and determines the rate of genetic drift and inbreeding (Waples 2022). Increasing *N*_*e*_ of the broodstock will also mitigate the Ryman–Laikre effect (Ryman and Laikre 1991), which can occur when hatchery individuals originating from broodstock with low *N*_*e*_ contribute disproportionately to reproduction, resulting in a reduction of the total effective size of a population (Christie, Marine, French, and Blouin 2012; Hagen et al. 2021; Hagen and Karlsson 2023; Waples et al. 2016). In this context, genetic monitoring is critical in recovery programs to monitor the contribution of stocked individuals to wild populations. Multiallelic markers such as microsatellites are particularly advantageous to perform parentage assignment (Vandeputte et al. 2021; Wenne 2023), to estimate *N*_*e*_ via the linkage disequilibrium method (Do et al. 2014), and to directly quantify the genetic diversity released into the wild (Almodóvar et al. 2020).

Reduced fitness of hatchery fish once released into the wild is a widely documented issue of supportive breeding (Araki et al. 2007; Bouchard et al. 2022; Jonsson et al. 2019; Roth et al. 2025). Recent studies have reported substantial differences in DNA methylation patterns between hatchery‐reared and wild fish (Koch et al. 2023), which may provide a mechanistic explanation for the reduced fitness of hatchery fish after release (Le Luyer et al. 2017; Rodriguez Barreto et al. 2019; Venney et al. 2025). Also, hatchery rearing alters the selective environment experienced by salmon, replacing natural selection with captivity‐associated selection, and can lead to rapid genetic changes, with measurable fitness consequences in the wild observed after a single generation (Christie, Marine, French, and Blouin 2012; Ford 2002; Frankham 2008). The resulting domestication‐related genetic changes may be transmitted across generations, reducing competitiveness and survival in natural environments (Christie et al. 2016; Farquharson et al. 2021; Garcia De Leaniz et al. 2007; Reed et al. 2015). Consequently, minimizing the number of generations in captivity and using exclusively wild broodstock can help mitigate those effects (Gilbey 2022; Lennox et al. 2021; Williams and Hoffman 2009).

Also, numerous release strategies have been implemented to reduce fitness‐related impacts, with juvenile stages ranging from eggs to smolts being released (Fraser 2016; Hagen and Karlsson 2023; Roth et al. 2025). Smolt release usually displays better survival and return rates than younger stages but can have lower reproductive success in the wild (Hagen and Karlsson 2023; Milot et al. 2013; Thériault et al. 2010). Releasing hatchery‐reared fish at the fry stage has multiple advantages, including lower rearing costs (Birnie‐Gauvin et al. 2018) and reduced artificial selection, while also circumventing the typically high mortalities observed in the wild between the egg and fry stages (0%–90% Dumas and Marty 2006). Differences in survival among release stages may be partly explained by microbiota recruitment mainly driven by the environment (Lavoie et al. 2018, 2021; Uren Webster et al. 2020). Given the high level of local adaptation in Atlantic salmon, rivers host distinct microbial communities that are acquired by juveniles and survival may be improved when acquisition occurs during earlier stages of development (Lavoie et al. 2021; Minich et al. 2020).

The Romaine watershed in eastern Québec provides a valuable case study for examining the impacts of a supportive breeding restoration program on two geographically proximate but genetically differentiated anadromous Atlantic salmon ( *Salmo salar* ) populations. This species progresses through several life stages: eggs are deposited in gravel at the bottom of rivers, fry (with yolk sacs) and then parr rear in freshwater, often for several years, before undergoing smoltification and migrating to the ocean as smolts. After one or more years at sea, adults return to their natal rivers to reproduce, completing their life‐cycle (Aas 2011). Following Hydro‐Québec's plan in the early 2000s to construct four dams along the Romaine River, environmental assessments revealed critically low numbers of breeders (Fontaine et al. 2000), prompting the implementation of a restoration program. Subsequent genetic analyses confirmed significant differentiation between salmon from the main Romaine River and its Puyjalon tributary (Dionne et al. 2008, *F*_ST_ = 0.031; Fauchet, Laporte, et al. 2026, *F*_ST_ = 0.099). Each population has been restored separately using wild‐caught smolts transferred to the Laboratoire Aquatique des Sciences Environnementales et Médicales (LARSEM) at Laval University to constitute population‐specific broodstocks. Eggs from both populations were incubated either under controlled conditions at LARSEM or at the Romaine River hatchery using local river water, and unfed vesiculated fry were used for stocking. This study aims to synthesize the demographic and genetic outcomes of a closely monitored 11‐year restoration program. Specifically, we aim to (1) evaluate the capacity of broodstocks to maintain genetic diversity in stocked individuals for both populations; (2) quantify the demographic and genetic impacts of stocking using parentage assignment; and (3) determine whether egg incubation using Romaine River water improves fry, parr, and smolt survival in both populations.

## 2 Materials and Methods

### 2.1 Broodstock Capacity to Maintain Genetic Diversity

#### 2.1.1 Sampling of Individuals

**FIGURE 1:**
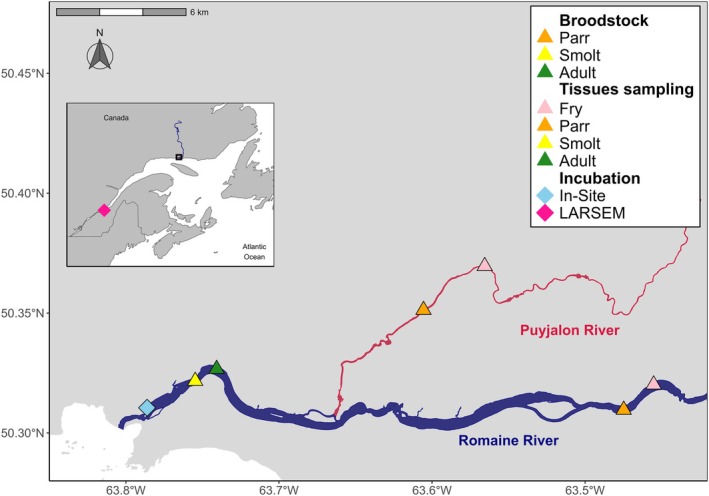
Map of the Romaine watershed (Québec, Canada) showing the study area with the Romaine River (blue) and its tributary Puyjalon (red). Triangles indicate sample sites of fry, parr, smolt and adult used for broodstock collection or tissue sampling for genetic analyses. Diamonds indicate incubation experiments sites comparing in situ and hatchery conditions.

This study was conducted in the Romaine and Puyjalon rivers in the Minganie region of Québec, Canada (Figure 1). Between 2013 and 2022, 16 adults, 546 smolts, and 164 parr were captured and transferred to the LARSEM to establish the broodstock. Smolts were captured using trap nets downstream of the river confluence, and parr as well as fry were collected using seine nets in their respective rivers (Figure 1). Adults were captured in 2013 using cages and a counting fence (Figure 1). Individuals were tagged with passive integrated transponder (PIT) tags, and fin clips were collected for genotyping.

#### 2.1.2 Genotyping and Population Assignment

DNA was extracted from tissue samples using the salt extraction method following Aljanabi (1997). Bidirectional sequencing was conducted on an Illumina MiSeq sequencer (V3 reagents, Illumina, San Diego, USA) to genotype all individuals with 52 microsatellite markers (Bradbury et al. 2018). These markers are part of a 101‐microsatellite panel, 70 to 130 base pairs in length with 10 to 15 repeats per locus. Sequences were quality‐filtered, and individual genotypes for the 52 selected markers were determined using MEGASAT (Zhan et al. 2017) with a minimum depth threshold of 16 sequences. Eleven markers with excessive missing data (> 15%) or displaying a single allele were removed (Supporting Information S1). The remaining 41 microsatellites were broadly distributed across genomic regions; therefore, capturing genome‐wide variation. The number of alleles per locus and F_ST_ per locus were calculated between the two reference populations from Puyjalon and Romaine rivers using the hierfstat R package (Goudet 2005) (Supporting Information S2). Individuals with more than 20% missing data were excluded from all analyses.

Reference populations for population assignment were the same as those used in Fauchet, Laporte, et al. (2026), with 132 individuals from the Romaine population and 169 from the Puyjalon. LARSEM individuals were assigned to their most likely population of origin using GeneClass (Piry et al. 2004) with the Bayesian criterion of Rannala and Mountain (1997) and a score threshold of 0.05. An individual was assigned to a given population only if its probability of being assigned to it was at least 10 times higher than that of being assigned to the other population. Individuals that did not meet this criterion were classified as “Undetermined” and removed from the dataset.

#### 2.1.3 Artificial Reproduction

Crosses were performed using a semi‐factorial design in which the eggs of each female were divided into three portions (or four for larger egg masses), with each portion fertilized by a single male, resulting in three or four families per female. Males and females were always paired according to their population of origin (Romaine or Puyjalon). All crosses were recorded in a database to ensure traceability and avoid repeating the same family crosses across years. To minimize crossing of closely related individuals, mean kinship was calculated for all potential breeders and UPGMA trees were generated based on the proportion of shared alleles for each population annually (more details are available in Supporting Information S3 and S4). Each individual could be used in reproduction for a maximum of four breeding years to maximize genetic variability of offspring throughout the stocking program.

#### 2.1.4 Effective Number of Breeders in the Broodstock

To assess the effective number of breeders *N*_*b*_ in the supportive breeding program, we used several complementary approaches based on demographic and reproductive data collected between 2014 and 2023. First, the number of males (*N*_*m*_) and females (*N*_*f*_) used for reproduction from each population and in each year from 2014 to 2023 were used to calculate the realized number of breeders (*N*_*b*_real) using the following formula (Wright 1931):

(1) $$N_{b}=\frac{4N_{m}N_{f}}{N_{m}+N_{f}}$$

To estimate the potential effective number of breeders (*N*_*b*_pot) in each population and year, we reconstructed the annual broodstock from hatchery inventory records under a theoretical scenario in which all individuals introduced into the captive broodstock survived to maturity and participated in reproduction. The timing of first potential reproduction was determined by life‐history stage at arrival: adults were assumed capable of reproducing in their arrival year, whereas smolts and parr were assumed to mature after 2 or 3 years and 3 or 4 years, respectively, reflecting maturation schedules observed under LARSEM conditions. Maturation was assigned separately to males and females, with 50% of individuals maturing at the earlier age and 50% at the later age within each sex. Once mature, individuals were assumed to reproduce annually for a maximum of four consecutive years. Based on this reconstructed broodstock and assuming an equal sex ratio among potential breeders, *N*_*b*_pot was calculated using Equation (1). This metric represents the maximum potential effective number of breeders achievable under the broodstock composition and does not account for mortality, non‐participation in reproduction, or variance in reproductive success. *N*_*b*_pot should therefore be interpreted as a theoretical upper bound, allowing comparison with *N*_*b*_real and *N*_*b*_var (calculated below) to highlight the effects of mortality, unequal reproductive success, and offspring survival on realized breeder contributions.

For each cohort and population between 2014 and 2023, we recorded the number of eggs produced by every parental pair in the supportive breeding program. These counts represent the potential reproductive contribution of each male and female to the offspring pool. Based on family size distributions, the mean (*k*) and variance (*V*_*k*_) in the number of offspring per male or female broodstock were recorded for each year. The number of breeders for each sex was estimated as follows:

(2) $$N_{b}=\frac{\mathrm{kN}-2}{k-1+\frac{V_{k}}{k}}$$

where *N* equals the number of broodstock males or females used for each cohort (Caballero 1994; Crow and Kimura 1970). The estimates for both sexes were then used to calculate *N*_*b*_var using Equation (1) but replacing *N*_*m*_ with *N*_*b*_ *m* and *N*_*f*_ with *N*_*b*_ *f*. Here, *N*_*b*_var reflects the true number of breeders assuming all eggs have equal survival probability. Since egg‐to‐fry survival data were available by family for the years 2020–2023, unlike in previous years when families were mixed during incubation, we also calculated *N*_*b*_stock for those years using Equations (1) and (2) with the number of offspring that survived until stocking. All four *N*_*b*_ estimates were compared to assess potential loss in effective number of breeders through the stocking process.

#### 2.1.5 Parentage Assignation and Genetic Diversity in the Broodstock

All individuals transferred at LARSEM after 2017, 2 years after the first stocking, were assigned based on their genotypes to potential parents using COLONY (Jones and Wang 2010) allowing male and female polygamy but excluding inbreeding. The full likelihood method was applied with high precision. Offspring and potential parent genotypes were provided to COLONY for assignment. For each analysis, offspring were grouped by capture year, and only potential parents capable of producing offspring in that year were included (Table 1). Among identified parent pairs, only those with a probability of 1 and recorded in the cross database were accepted.

**TABLE 1:** Number of potential parents (*N*_Dad_ and *N*_Mom_) used in Colony for each year of sampled individuals. *N* (LARSEM) indicates the number of fish each year in the LARSEM. *N* (tissue sampling) indicates the number of fish sampled this given year.

Year	Stage	*N* (LARSEM)	*N* (tissue sampling)	*N*_Dad_ (LARSEM)	*N*_Mom_ (LARSEM)
2021	Adult	0	11	132	177
2022	Adult	0	35	133	178
2017	Smolt	13	248	126	162
2018	Smolt	2	99	131	165
2019	Smolt	36	229	131	165
2020	Smolt	88	326	132	177
2021	Smolt	120	291	133	178
2022	Smolt	5	478	159	213
2023	Smolt	0	318	199	279
2024	Smolt	0	303	270	359
2018	Parr	25	0	131	165
2019	Parr	18	0	132	177
2020	Parr	29	0	130	181
2021	Parr	87	0	159	213
2023	Parr	0	116	270	359
2024	Parr	0	97	314	407
2020	Fry	0	192	199	279
2021	Fry	0	180	270	359
2023	Fry	0	294	314	407

Expected and observed heterozygosity (*H*_*E*_ and *H*_*O*_), allelic richness (*A*_*R*_), and the inbreeding coefficient (*F*_IS_) according to Weir and Cockerham (1984) were then estimated for each population annually with the R package hierfstat (Goudet 2005). To evaluate uncertainty, a nonparametric bootstrap with 1000 replicates over individuals was performed using the R package boot (Canty and Ripley 1999). For F_IS_, significance was assessed by examining whether confidence intervals overlapped zero. Mean kinship (MK) between individuals was also estimated for each year and population broodstock using the *related* R package (Pew et al. 2015), allowing for inbreeding. Pairwise relatedness coefficients were first estimated using the maximum‐likelihood TrioML estimator (Wang 2007), which accounts for both genotyping error (set at 1%) and potential inbreeding. Individual MK was then calculated as the mean relatedness coefficient between each individual and all other individuals within the same population. Population‐level MK was summarized as the mean ± standard error of individual values. To evaluate the influence of population on individual MK while accounting for temporal structure, a linear mixed‐effects model was fitted with individual MK as the response variable, population as a fixed effect, and year as a random effect using the *lme4* package.

### 2.2 Demography and Genetic Impact of Stocking

#### 2.2.1 Stocking and Sampling of Individuals

From 2015 to 2023, offspring produced at LARSEM were stocked in their respective rivers as unfed fry (730,452 fry were stocked in the Puyjalon River and 649,745 in the Romaine River). Following stocking, 677 fry, 217 parr, 2315 smolts, and 46 adults were sampled between 2017 and 2024. Fry were captured using electrofishing or seine nets and euthanized using an MS‐222 bath in 2020, 2021, and 2023. Sampling was conducted annually in early September in the upper reaches of each river (Figure 1). A total of 172 fry were captured in the Puyjalon River and 505 in the Romaine River. In 2023 and 2024, parr were sampled using the same methods in different areas of both rivers: 38 in the Puyjalon River and 179 in the Romaine River. The 2316 smolts were captured using trap nets in the lower Romaine River between 2017 and 2023, fin‐clipped, and scale‐sampled before release. Representative sampling of smolts was achieved by selecting individuals annually based on their distribution across capture dates and size classes, ensuring the subset reflected overall smolt captures in the Romaine River. Smolts were aged following the protocol using scales described in Aubé‐Maurice et al. (2019). The 46 adults were captured in 2021 and 2022 in the Romaine River (Figure 1) using fly fishing, fin‐clipped, and released. DNA was extracted and individuals were genotyped, assigned to their population of origin, and assigned to potential parent pairs as described in Sections 2.1.2 and 2.1.5 (Table 1).

#### 2.2.2 Demography in the Watershed

##### 2.2.2.1 Relative Contribution of Romaine and Puyjalon Populations and Stocked Fish in Smolts

Using smolts sampled from 2017 to 2024, the relative contribution of the two populations (Romaine and Puyjalon) across years was quantified. Sex ratios were then compared between populations and life stages using captured fry, parr, and smolts. Differences in sex ratio were tested using a generalized linear mixed model (GLMM) with the lme4 R package (Bates et al. 2015) with a binomial error distribution and logit link. The response variable was sex (male = 1, female = 0) and fixed effects included population, life stage (Fry, Parr, Smolt), and their interactions, while year was included as a random intercept to account for repeated sampling across years. Differences in relative contributions of stocked individuals between populations after 2019 were tested using a GLMM with stocked (stocked = 1, not stocked = 0) as the response variable, population as a fixed effect, and year as a random intercept to account for repeated sampling across years.

##### 2.2.2.2 Smolt Abundance Estimate

The abundance of smolts was estimated with Capture‐Mark‐Recapture (CMR) methods using the Petersen (1896) estimator modified by Chapman (1951):

(3) $$N=\frac{\left(M+1\right)\left(C+1\right)}{\left(R+1\right)}$$

where *M* is the number of marked smolts, *C* is the total captures, and *R* is the recaptures. The CMR design was based on a single sampling site in the Puyjalon River, where smolts were captured in trap nets, marked, and released upstream of the capture zone to allow mixing with the population before potential recapture during subsequent daily net checks. Marking consisted of partial adipose fin clipping, allowing rapid and visible individual identification. Confidence intervals were calculated following Zar (1984), which accounts for variability in recaptures, captures, and their ratio. Smolt abundance with confidence intervals was first estimated in the Puyjalon River. Then it was extrapolated to the whole watershed using the genetic assignment ratio where sampled smolts were captured, where both populations are mixed (Aubé‐Maurice et al. 2019, 2020, 2021, 2022, 2024a, 2024b, 2025; WSP 2014, 2015, 2016, 2017, 2019). To evaluate whether abundance changed significantly through time, a linear model for each population was fitted.

##### 2.2.2.3 Nest Counting

Prior to nest counts, spawning grounds in the main channel of the Romaine River and the Puyjalon River were monitored to document spawning progression and ensure counts were conducted only after spawning was completed. Nest counts were consistently performed between late October and mid‐November by snorkeling and diving. Salmon nests were identified using the following criteria: (1) an oblong depression oriented with water flow, (2) dimensions of 0.5–1.5 m in length and 0.3–1 m in width, and (3) substrate with a paler color than surrounding material, free of fine particles and sand between larger granules, indicating recent cleaning and rearrangement. Nest counts were conducted in 2003 and 2004, and annually between 2010 and 2024. Further methodological details are available in technical reports (Aubé‐Maurice et al. 2019, 2020, 2021, 2022, 2024, 2025; Belles‐Isles et al. 2004; GENIVAR 2005, 2011, 2012, 2013; WSP 2014, 2015, 2016, 2017, 2019). Temporal trends in nest abundance in the two rivers were tested using a negative binomial generalized linear model (GLM) including year and river as fixed effects and their interaction with the glm.nb() function from the MASS package in R (Venables and Ripley 2002).

##### 2.2.2.4 Adults Return

Adult Atlantic salmon returning to the watershed were enumerated using a floating articulated barrier equipped with the automated counting system IchtyoS developed by WSP (https://www.wsp.com/en‐ca). Each monitoring year, the barrier was installed at kilometre 7.3 of the river, downstream of the confluence with the Puyjalon River, a site selected in 2010 due to its favorable bathymetric profile and flow conditions. The IchtyoS system classifies fish into three size categories: < 50, 50–63, and > 63 cm, which fit the size distribution of Atlantic salmon. Individuals larger than 63 cm were considered multi‐sea‐winter salmon, whereas those between 50 and 63 cm were considered one‐sea‐winter salmon. Fish smaller than 50 cm were considered juveniles or likely other species. As no genetic monitoring was performed, returning individuals could not be assigned to either the Romaine or Puyjalon populations.

Monitoring was performed in 2010, 2013, 2015, 2018, 2021, and 2024. Counts from 2010 were likely underestimated because the barrier did not completely block fish passage in its first year. To evaluate temporal trends in Atlantic salmon abundance, a linear model (LM) was fitted to the total number of adults over time, excluding 2010 due to incomplete data. This model tested for a linear increase or decrease in annual adult counts. A binomial generalized linear model (GLM) was then used to assess trends in sea‐age composition, treating the counts of one‐sea‐winter (1SW) and multi‐sea‐winter (MSW) adults as a binomial response. The GLM included all years, including 2010, and modeled changes in the proportion of 1SW relative to MSW adults over time.

##### 2.2.2.5 Reproduction of Stocked Fish

To test whether hatchery fish returned as adults and reproduced, the 11 adults sampled in 2021 were tested as potential parents for parr captured in 2023 and smolts caught in 2024, while the 35 adults sampled in 2022 were tested as potential parents for the fry and parr respectively sampled in 2023 and 2024. Smolts from 2017 to 2024 were tested as potential parents of fry, parr, and smolts sampled from 2021 to 2024 that were not already assigned to a hatchery parent pair. Potential parents were tested according to the biological possibility of having produced offspring of a given stage at a given year to avoid impossible parent pairs (Table 2).

**TABLE 2:** Number of potential parents (*N*_Dad_ and *N*_Mom_) used in Colony for each year of sampled individuals to find hatchery individuals that reproduced.

Stage and year tested	*N*	Year	Stage	*N*_Dad_	*N*_Mom_
Parr 2023/Smolt 2024	317	2021	Adult	5	6
Fry 2023/Parr 2024	212	2022	Adult	18	17
Smolt 2021	251	2017	Smolt	93	155
Smolt 2022	371	2017–18	Smolt	131	216
Smolt 2023	218	2017‐18‐19	Smolt	213	363
Smolt 2024	241	2017‐18‐19‐20	Smolt	349	553
Parr 2023	76	2017‐18‐19‐20	Smolt	349	553
Parr 2024	53	2018‐19‐20‐21	Smolt	384	557
Fry 2020	136	2017–18	Smolt	131	216
Fry 2021	139	2017‐18‐19	Smolt	213	363
Fry 2023	159	2018‐19‐20‐21	Smolt	384	557

Parent‐offspring assignments identified both complete pairs and single parents (mother or father). Offspring and parent population was checked for concordance and only parents assigned with probability of 1 were retained. For smolts as potential offspring, age at smoltification concordance was also verified. Chi‐square tests were performed to test whether the proportion of adults and smolts from wild versus hatchery origin differed between potential reproducers and actual reproducers. Fisher's exact test was used when sample size was less than 5.

#### 2.2.3 Estimation of the Ryman Laikre Effect

The Ryman–Laikre effect describes a counterintuitive situation in which a supportive breeding (or stocking) program can reduce N_e_, even when the total number of individuals increases. To test for this effect, the effective number of breeders per cohort for wild smolts (*N*_*w*_) was estimated using NeEstimator (v2.0.1; Do et al. 2014). The linkage disequilibrium (LD) method was used with 0.05 as the lowest allele frequency, and confidence intervals were calculated by jackknifing over loci. The effective number of breeders per cohort for hatchery smolts (*N*_*c*_) was estimated using both the LD method (*N*_*c*_LD) as well as a pedigree‐based approach (*N*_*c*_ped) as follows: after assigning hatchery smolts to known broodstock pairs, the mean (*k*) and variance (*V*_*k*_) in number of offspring per male and female broodstock for each year and the number of breeders for each sex (*N*_*b*_ *m* and *N*_*b*_ *f*) were estimated using Equation (2). These estimates were then used to calculate *N*_*c*_ped with equation (1). *N*_*c*_LD and *N*_*c*_ped were then compared using a linear regression. Using the equation presented in Ryman and Laikre (1991), the effective number of breeders for hatchery and wild smolts was calculated as:

(4) $$\frac{1}{N_{e}}=\frac{x^{2}}{N_{c}}+\frac{{\left(1-x\right)}^{2}}{N_{w}}$$

where *N*_*c*_ (*N*_*c*_LD or *N*_*c*_ped) and *N*_*w*_ are the effective number of hatchery and wild breeders, respectively, and *x* is the proportion of hatchery fish in each smolt cohort. Finally, the ratio between *N*_*e*_ and *N*_*w*_ was calculated to assess the Ryman–Laikre effect. A ratio below 1 indicates that hatchery supplementation reduced the effective population size.

#### 2.2.4 Genetic Diversity

Genetic diversity indices (*H*_*E*_, *H*_*O*_, *A*_*R*_, and *F*_IS_) were calculated as described in Section 2.1.5 for each cohort of each population, separately for hatchery and wild origin smolts. Cohorts with fewer than 10 fish were excluded. Linear models were fitted for each index to compare populations and hatchery versus wild smolts. Genetic differentiation between the Romaine and Puyjalon populations across cohorts was quantified using Nei's pairwise *F*_ST_ estimator (Nei 1978) implemented in the hierfstat R package (Goudet 2005). Confidence intervals were estimated using 1000 bootstraps.

Finally, we contrasted the relative contribution of captive‐bred individuals to allelic richness with that of wild individuals by calculating the total number of alleles across all loci found among progeny assigned to either wild pairs or captive‐bred pairs in incremental subsets of all sampled offspring. Individuals were randomly subsampled from 50 to 1500 (or the maximum number available) in steps of 50, with 1000 replicates per step. The difference in the number of alleles between the two groups was represented with a Loess regression of the mean total number of alleles for each sample size, along with the 95% distribution of values.

### 2.3 Comparison of Survival for Each Incubation Treatment

#### 2.3.1 Egg Incubation

Starting in 2019, eggs obtained from captive breeding, from both the Romaine and Puyjalon populations were incubated under two distinct treatments to evaluate survival success (Table 3). The Optimized Prophylactic Treatment (OPT) was conducted at the LARSEM facility (Figure 1), where fertilized eggs were maintained under controlled conditions, including water recirculation, regulation of pH, oxygen, and temperature, UV sterilization, and regular removal of dead eggs. In contrast, a Natural Microbiota Treatment (NMT) was implemented at the Romaine River hatchery (Figure 1, in‐site incubation), where eggs were incubated in untreated river water with the sole intervention consisting of the regular removal of dead eggs. For both populations, families were randomly assigned to one of the two treatments immediately after fertilization, and incubation continued from the fertilized egg stage to vesiculated fry, just prior to stocking. This design ensured parentage traceability for survival analyses but may also introduce family‐specific survival bias.

**TABLE 3:** Number of unfed fry stocked between 2015 and 2023 in both rivers. Treatment OPT indicates optimal prophylactic treatment and MPT indicates minimal prophylactic treatment.

	Romaine		Puyjalon	
2015	0		22,000	
2016	9546		15,711	
2017	116,073		112,493	
2018	72,238		90,029	

Treatment	OPT	MPT	OPT	MPT
2019	62,267	40,905	85,362	32,343
2020	59,959	59,430	72,602	67,562
2021	21,139	35,869	46,020	56,325
2022	20,055	43,096	47,952	23,026
2023	38,765	48,493	38,427	42,600

#### 2.3.2 Egg‐To‐Fry Survival

The effect of treatment type on egg‐to‐fry survival was evaluated using family survival data from 2020 to 2023. The dataset included 513 crosses with known total offspring and number of surviving offspring just before stocking. A generalized linear mixed‐effects model (GLMM) with a binomial distribution was fitted using the glmer function from the lme4 package (Bates et al. 2015). The model tested the effects of treatment type, population of origin, and their interaction on offspring survival, while accounting for annual variation by including year as a random effect. Estimated marginal means (predicted survival probabilities) and pairwise contrasts were obtained using the emmeans package (Lenth and Piaskowski 2017), with results back‐transformed to the probability scale.

#### 2.3.3 Treatment Survival

All sampled fish assigned to families stocked between 2019 and 2023 were used to compare the two incubation treatments. This includes assigned individuals monitored in the wild (smolts, parr, and fry) as well as assigned individuals transported at LARSEM. Assigned fry, parr, and smolts were analyzed separately to compare the effect of treatment on survival across stages for the two populations. For each stage, population, and year, the number of individuals assigned to each treatment was calculated, along with the number of stocked fry.

To assess differences in relative survival at all three stages, three generalized linear mixed models (GLMMs) were fitted using the glmer function from the lme4 package in R. Survival (the number of surviving versus dead individuals) was modeled as a binomial response variable with a logit link. The model included treatment type, population (Romaine vs. Puyjalon), and their interaction as fixed effects. The interaction term tested whether the effect of treatment on survival differed between populations. Year was included as a random effect to account for interannual variability in environmental conditions and experimental settings and to avoid pseudoreplication across cohorts. Odds ratios and confidence intervals were calculated to compare relative survival under the two incubation treatments using the emmeans package (Lenth and Piaskowski 2017).

## 3 Results

### 3.1 Broodstock Capacity to Maintain Genetic Diversity

#### 3.1.1 Population Assignment of Broodstock Individuals

Among the 726 individuals that arrived at LARSEM to constitute the broodstock, 5 parr with more than 20% missing data were removed from the analyses and potential reproduction. Two adults and two smolts could not be confidently assigned to either of the two populations and were also removed. After population assignment, the broodstock included 446 individuals from Puyjalon (266 females and 180 males) and 271 from Romaine (141 females and 130 males) (Supporting Information S5).

#### 3.1.2 Artificial Reproduction

In total, 1414 crosses were performed between 2014 and 2023, 751 families for the Romaine population and 663 for the Puyjalon population. Romaine families were composed of 98 unique reproducing females (69.5% of all broodstock females) and 74 unique reproducing males (56.9% of all broodstock males). Puyjalon families were composed of 156 unique reproducing females (58.6% of all broodstock females) and 82 reproducing males (45.6% of all broodstock males).

#### 3.1.3 Effective Number of Breeders

**FIGURE 2:**
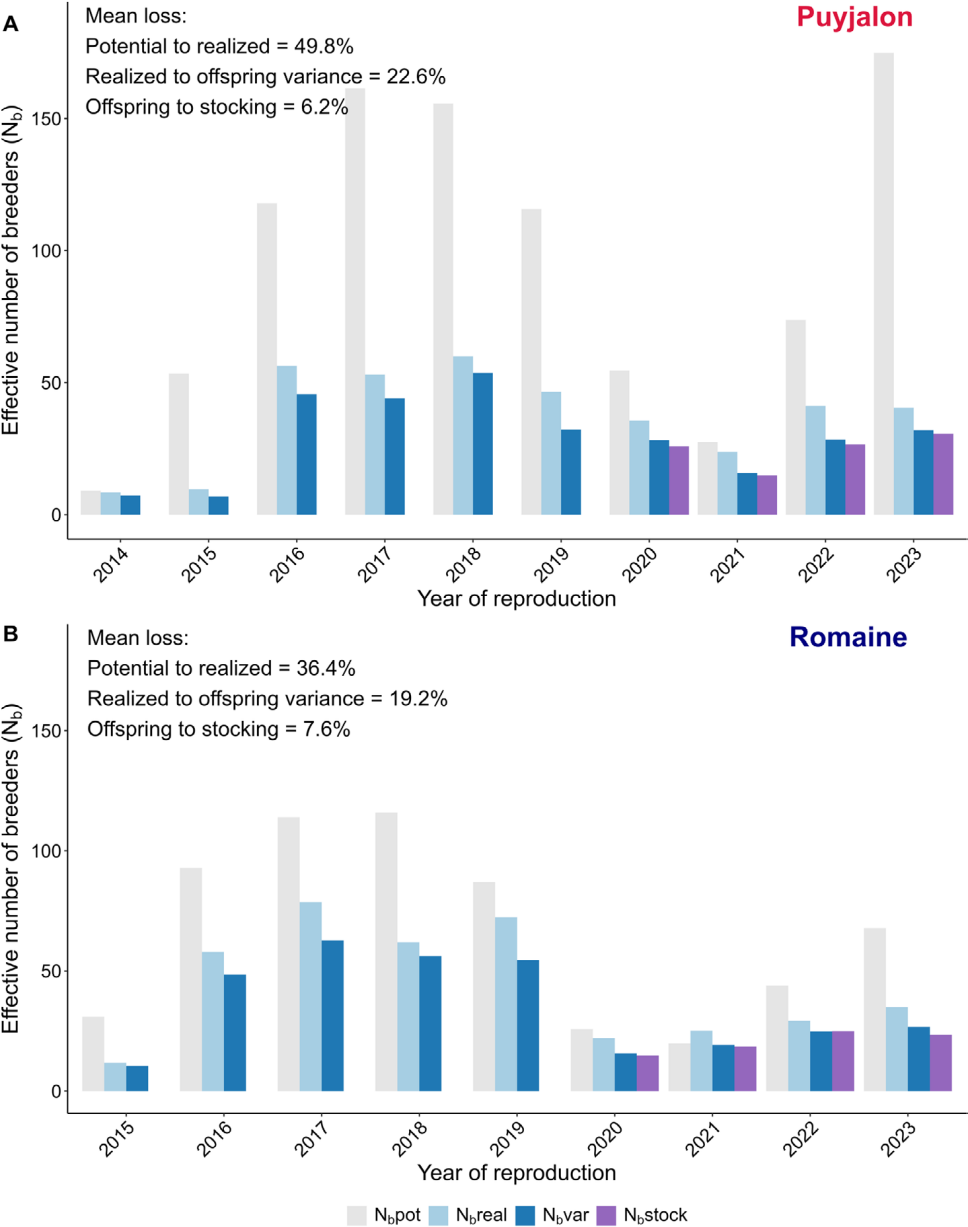
Potential (*N*_*b*_pot) and realized (*N*_*b*_real) effective number of breeders in captivity calculated with only sex ratio data (Equation 1), as well as *N*_*b*_ calculated with offspring variance (*N*_*b*_var) (Equations 1 and 2) and just before stocking (*N*_*b*_stock) from 2015 to 2023; (A) in the Puyjalon population; (B) in the Romaine population.

The average potential (*N*_*b*_pot) and realized (*N*_*b*_real) number of breeders did not differ significantly between populations. However, differences were more pronounced for *N*_*b*_pot (average *N*_*b*_pot across years for Puyjalon: 94.4 ± 18.6; Romaine: 66.5 ± 12.6) than for *N*_*b*_real, for which values were more similar between populations (average *N*_*b*_real across years for Puyjalon: 37.5 ± 5.8; Romaine: 43.8 ± 8.1). Average loss between *N*_*b*_pot and *N*_*b*_real was 49.8% in the Puyjalon population and 36.4% in the Romaine population (Figure 2). Both estimates followed the trend of broodstock renewal, which declined sharply after 2014 (Supporting Information S5). In the Romaine broodstock in 2021 *N*_*b*_real was higher than *N*_*b*_pot because frozen male sperm was used to enhance the number of breeders. The use of offspring variance to calculate *N*_*b*_var substantially reduced *N*_*b*_, by 22.6% in the Puyjalon population and by 19.2% in the Romaine population (average *N*_*b*_var across years for Puyjalon: 29.4 ± 5.0; Romaine: 35.4 ± 6.8; Figure 2). Variance in offspring was substantially higher for males than females in both populations and remained relatively stable for females but increased drastically for males after 2018 (Supporting Information S6), leading to a reduced *N*_*b*_var in both populations especially after 2018. Between 2020 and 2023, family specific egg‐to‐fry survival was highly variable (Supporting Information S6) with an average survival rate of 70.9% ± 1.41%, ranging from 0.0% to 99.2%. After accounting for offspring survival during the period of 2020–2023, the average loss of *N*_*b*_ between *N*_*b*_var and *N*_*b*_stock was 6.2% in the Puyjalon population and 7.6% in the Romaine population (average *N*_*b*_stock across years for Puyjalon: 24.5 ± 3.4; Romaine: 20.4 ± 2.3; Figure 2).

#### 3.1.4 Parentage Assignment and Genetic Diversity in the Broodstock

**FIGURE 3:**
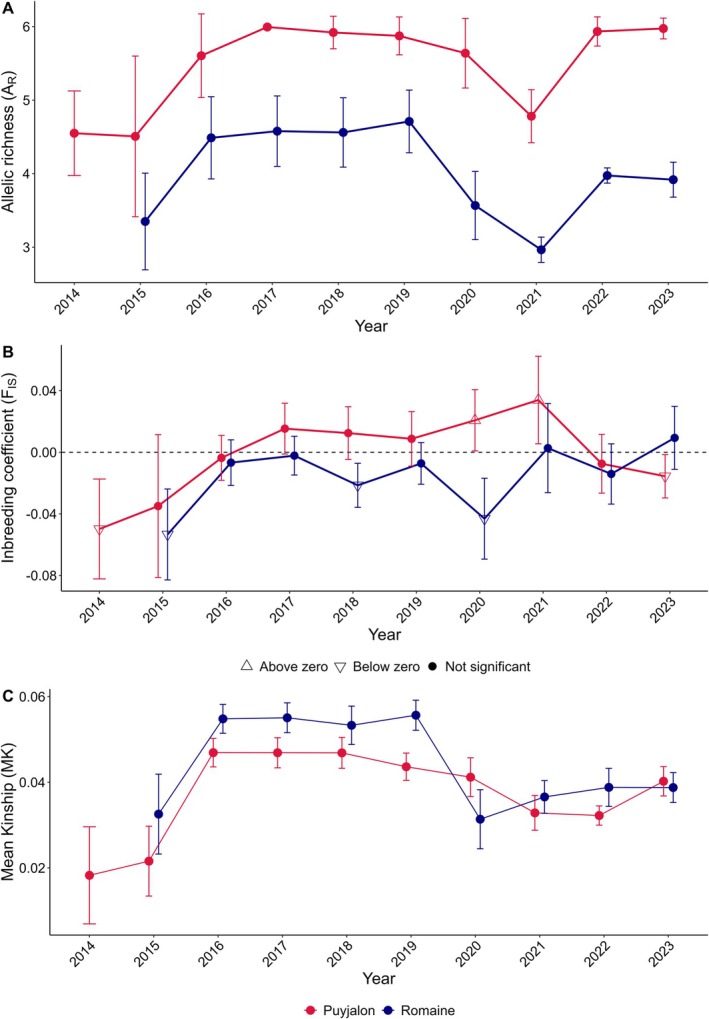
(A) Evolution inside the broodstock of: allelic richness (*A*_*R*_); (B) inbreeding coefficient; upward triangles denote significant heterozygote deficits (*F*_IS_ > 0), downward triangles denote significant heterozygote excesses (*F*_IS_ < 0), and filled circles denote non‐significant *F*_IS_ estimates. (C) Mean Kinship (MK).

In the Romaine population, 62 parr and 12 smolts were assigned to previous crosses of the broodstock, while 8 parrs and 29 smolts were assigned to previous crosses of the broodstock for the Puyjalon. The percentage of assigned individuals that reproduced ranged from 4.2% in Puyjalon 2021 to 55.6% in Romaine 2021 (Supporting Information S7A). Average expected heterozygosity (*H*_*E*_) between 2014 and 2023 was 0.56 ± 0.01 in the Puyjalon broodstock and 0.53 ± 0.01 in the Romaine broodstock. Observed heterozygosity showed similar differences between rivers (average *H*_*O*_ in Puyjalon: 0.56 ± 0.01 and in Romaine: 0.54 ± 0.01) (Supporting Information S7B,C). Allelic richness showed clear differences between populations and temporal variation across years (Figure 3A): in Puyjalon, mean *A*_*R*_ values ranged from 4.55 (2015) to 5.99 (2017), with relatively low annual variation (mean ± SD = 5.48 ± 0.39) while the Romaine population displayed lower allelic richness, with mean values ranging from 2.97 (2021) to 4.71 (2019) (mean ± SD = 4.01 ± 0.39) and greater temporal variation, with particularly low values in 2020–2021.

In the Puyjalon population, *F*_IS_ values fluctuated slightly around zero between 2014 and 2023 (mean ± SD = −0.002 ± 0.023). Significant negative values were observed in 2014 and 2023, suggesting a slight heterozygote excess, whereas slightly positive and significant values were detected in 2020 and 2021, suggesting inbreeding (Figure 3B). In the Romaine population, values were almost always negative, ranging from −0.058 to 0.009 (mean ± SD = −0.015 ± 0.020), suggesting a mild excess of heterozygotes across years, with significant negative values detected in 2015, 2018, and 2020 (Figure 3B). Mean kinship ranged from 0.018 in 2014 to 0.047 in 2016 in the Puyjalon broodstock (mean ± SD = 0.037 ± 0.004), and between 0.031 in 2020 and 0.056 in 2019 in the Romaine broodstock (Figure 3C). The mixed‐effects model revealed a small but significant effect of population on individual MK (*χ* ^2^ = 3.07, *p* < 0.002), with Romaine individuals exhibiting slightly higher mean kinship values than those from Puyjalon (fixed effect estimate = 0.0059 ± 0.0019 SE). Random year effects were weak (variance = 0.0086), indicating that temporal variation contributed minimally compared with among‐population differences.

### 3.2 Demographic and Genetic Impact of Stocking

#### 3.2.1 Population and Parental Assignment of Sampled Individuals

Among the 677 fry sampled in the wild between 2020 and 2023, six had more than 20% missing data (one sampled in the Puyjalon and five in the Romaine). Of the remaining fry, 231 were assigned confidently to the Puyjalon and 434 to the Romaine, while six individuals could not be assigned confidently to any population and were removed from subsequent analyses. Parentage assignment identified 53 individuals among Puyjalon fry (22.9%) and 184 individuals among Romaine fry (42.4%) assigned to the LARSEM broodstock. Among the 217 parrs sampled between 2020 and 2023, four had more than 20% missing data (all sampled in the Romaine). Of the remaining parr, 42 were assigned confidently to the Puyjalon and 169 to the Romaine, while two individuals could not be assigned confidently to any population and were removed from subsequent analyses. Parentage assignment identified 10 individuals among Puyjalon parrs (23.8%) and 77 individuals among Romaine parrs (45.6%) assigned to the LARSEM broodstock. Among the 2316 smolts sampled between 2017 and 2024, 37 had more than 20% missing data. Of the remaining smolts, 1934 were assigned confidently to the Puyjalon population and 327 to the Romaine population, while 18 individuals could not be assigned confidently to any population and were removed from all following analysis. Parentage assignment identified 354 individuals among Puyjalon smolts (18.3%) and 74 individuals among Romaine smolts (22.7%) assigned to the LARSEM broodstock. Finally, among the 46 adults sampled in 2021 and 2022, none had more than 20% missing data. Twenty‐seven adults were assigned confidently to the Puyjalon population and 18 to the Romaine population, while one individual could not be assigned confidently to any population and was removed from subsequent analyses. Parentage assignment identified 6 individuals among Puyjalon adults (22.2%) and 6 individuals among Romaine adults (33.3%) assigned to the LARSEM broodstock.

#### 3.2.2 Demography in the Watershed

##### 3.2.2.1 Relative Contribution of Romaine and Puyjalon Populations and Stocked Fish in Smolts

Between 2017 and 2024, the relative contribution of the two populations was highly unbalanced, with Puyjalon representing on average 85.2% of sampled smolts (range: 79.7%–90.2%, SE = 1.4%) and Romaine accounting for 14.8% (range: 9.8%–20.3%, SE = 1.5%). The GLMM suggested a tendency for sex ratio to vary with life stage (*χ* ^2^ = 4.82, df = 2, *p* = 0.090), although this effect was not statistically significant. Smolts from Puyjalon were less frequently males (predicted probability = 0.36, 95% CI: 0.34–0.39) compared to fry (0.44, 95% CI: 0.37–0.51) or parr (0.51, 95% CI: 0.36–0.65). In contrast, the sex ratio among Romaine individuals was more balanced across life stages, with predicted male proportions of 0.49 (95% CI: 0.44–0.54) for fry, 0.50 (95% CI: 0.43–0.58) for parr, and 0.50 (95% CI: 0.44–0.56) for smolts. The interaction between population and stage was marginally supported (*p* = 0.090), suggesting that stage‐specific differences in sex ratio were more pronounced in Puyjalon than in Romaine.

**FIGURE 4:**
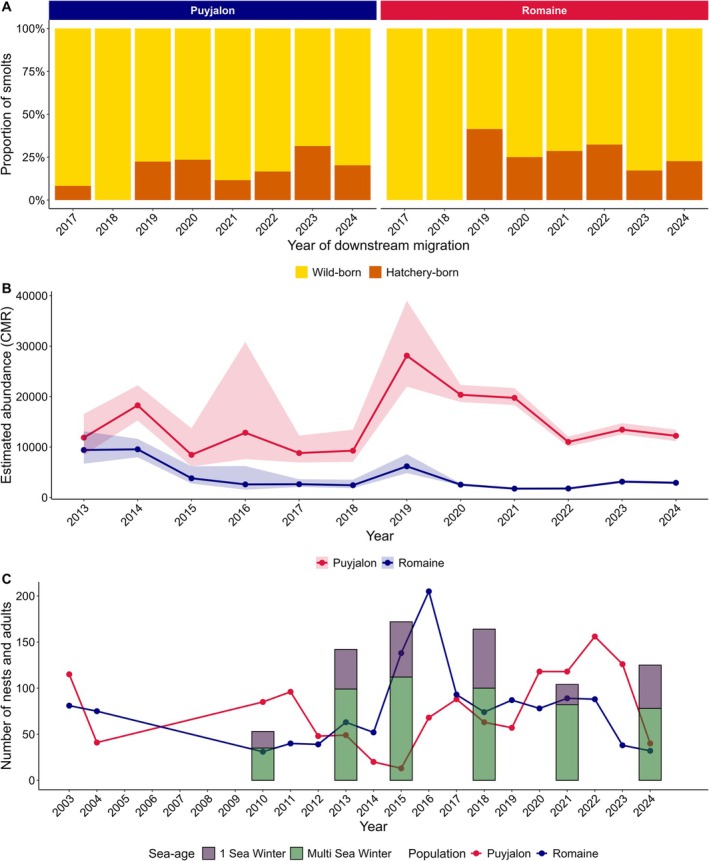
(A) Evolution of the proportion of smolt caught in the wild that are hatchery‐born individuals and wild‐born; (B) number of hatchery and wild smolt estimated with CMR, 95% confident intervals indicated with shaded area; (C) number of nests (lines) and number of adults (barplot) at the migration barrier each year in each river.

From 2019 to 2024, an average of 21.0% of sampled Puyjalon smolts (range: 11.6%–31.5%, SE = 2.8%) and 27.9% of Romaine smolts (range: 17.2–41.4, SE = 3.4%) originated from the LARSEM broodstock. The GLMM indicated a significant higher proportion of stocked fish in the Romaine population compared to Puyjalon (log‐odds = 0.304, SE = 0.150, *p* = 0.043). The variance among years was low (*σ* ^2^ = 0.065), indicating relatively minor year‐to‐year variation (Figure 4A).

##### 3.2.2.2 Smolt Abundance Estimate

Across the 2013–2024 period, mean CMR (Chapman‐modified Petersen estimator) estimated abundance was substantially higher in Puyjalon (14,538 ± 1709) than in Romaine (4065 ± 803, Figure 4B). For Romaine, CMR estimates showed a significant decline over time, with an average decrease of approximately 504 individuals per year (*p* = 0.021, *R* ^2^ = 0.43). In contrast, Puyjalon abundance remained relatively stable through time (slope = +233 fish year^−1^, *p* = 0.66, *R* ^2^ = 0.02).

##### 3.2.2.3 Nest Counting

Across the 2003–2024 time series, the mean number of nests was nearly identical between populations, with Puyjalon averaging 76.5 ± 9.8 nests (SE) and Romaine averaging 76.6 ± 10.5 nests (SE) over 17 monitored years (Figure 4C). The highest counts were observed in 2016 for Romaine (205 nests) and in 2022 for Puyjalon (156 nests), while the lowest counts were recorded in 2015 in both populations (13 nests in Puyjalon and 31 in Romaine). The negative binomial regression revealed no significant difference in the number of nests between populations (*z* = 0.47, *p* = 0.63), no temporal trend in nest counts (*z* = 0.84, *p* = 0.40), nor a significant difference in trend between populations (interaction term: *z* = −0.48, *p* = 0.63).

##### 3.2.2.4 Adult Returns

Between 2010 and 2024, the number of adult Atlantic salmon returning in the watershed (no genetic data for those adults) ranged from 53 to 172 individuals, with a mean of 126.7 ± 17.9 (SE) (Figure 4C). The number of 1SW adults ranged from 18 to 64, and MSW adults from 35 to 112, yielding an average 1SW ratio of 0.33 ± 0.03 (SE). The linear model excluding 2010 indicated no significant trend in total adult abundance over time (slope = −3.88 ± 2.86, *t* = −1.36, *p* = 0.27, *R* ^2^ = 0.27). Similarly, the binomial GLM including all years showed no significant temporal trend in the proportion of 1SW versus MSW adults (year coefficient = 0.002 ± 0.018, *z* = 0.10, *p* = 0.92), indicating that the relative sea‐age composition did not change over time.

##### 3.2.2.5 Reproduction of Stocked Fish

Parentage assignment detected 55 offspring assigned to 16 unique adult parents, including two offspring with an inferred father and an inferred mother. More specifically, among Puyjalon offspring, six were assigned to five unique wild parents and five were assigned to two unique hatchery‐born parents (stocked in 2017 and 2018). Among Romaine offspring, 26 were assigned to six unique wild parents and 19 were assigned to three unique hatchery‐born parents (stocked in 2017 and 2018). A chi‐squared test revealed no differences in the proportion of wild and hatchery adult Romaine individuals that returned in 2021 and 2022 (*N* = 46) and the proportion of adults that were assigned to offspring in our dataset (*χ* ^2^ = 0.092, *p* = 0.76). The Fisher exact test on the Puyjalon population produced similar results (OR = 2.82, *p* = 0.238). Parentage assignment detected 46 offspring assigned to 29 unique smolt parents but no complete mate pair was identified. More specifically, among Puyjalon offspring, 19 were assigned to 14 wild smolts and three were assigned to two hatchery smolts. In Romaine offspring, 21 were assigned to 10 wild smolts and three were assigned to three hatchery smolts. No statistical tests were conducted due to small sample size.

#### 3.2.3 Estimation of the Ryman Laikre Effect

Sample sizes for the 2016 Puyjalon cohort (*N* = 3) and the 2019 and 2021 Romaine cohorts (*N* = 10 and 11, respectively) were too low to calculate the number of breeders for hatchery smolts using the LDNe method with NeEstimator (*N*_*c*_LD), and only the pedigree‐based method was used (N_c_ped; Equation 4). For all other cohorts, both *N*_*c*_LD and *N*_*c*_ped were calculated and were highly correlated (*R* ^2^ of 0.78). Therefore, only estimates of *N*_*e*_ based on *N*_*c*_ped (using Equation 1 and 2) are presented (Table 4, but see Supporting Information S8 for more details including confidence intervals for *N*_*c*_LD and *N*_*w*_). *N*_*c*_ped varied between 3.73 and 41.90 for the Puyjalon population with an average of 20.58 (±4.67) and between 9.12 and 25.20 for the Romaine population with an average of 17.54 (±2.85) (Table 4). The number of breeders for wild smolts (*N*_*w*_) varied between 24.2 and 75.4 for the Puyjalon population with an average of 49.13 (±5.85) and between 21.1 and 35.7 for the Romaine population with an average of 28.48 (±2.78). For all cohorts of the two populations, the final *N*_*e*_ was always higher than *N*_*w*_, showing no diminution of effective size in the watershed due to supplementation.

**TABLE 4:** Number of wild (wild) and hatchery (hatchery) smolts genotyped, proportion of hatchery smolts (*x*), the effective number of wild smolts (*N*_*w*_), the effective number of hatchery smolts calculated with the pedigree methods (*N*_*c*_ [ped]), the total effective population size as derived from (4) (*N*_*e*_ [ped]), a measurement of whether stocking caused a decrease in the total effective population sizes (*N*_*e*_ [ped]/*N*_*w*_).

Population	Cohort	Wild	Hatchery	*x*	*N*_*w*_	*N*_*c*_ (ped)	*N*_*e*_ (ped)	*N*_*e*_(ped)/*N*_*w*_
Puyjalon	2015	94	18	0.16	44.7	5.91	49.68	1.11
	2016	36	3	0.08	43.4	3.73	47.13	1.09
	2017	189	104	0.35	75.4	41.9	117.30	1.56
	2018	217	26	0.11	69	29	83.67	1.21
	2019	215	48	0.18	52.6	31.4	72.64	1.38
	2020	145	85	0.37	24.2	21.4	43.85	1.81
	2021	108	41	0.28	37.5	19.6	55.95	1.49
	2022	112	29	0.21	46.2	11.7	57.90	1.25
Romaine	2018	27	10	0.27	24.6	25.2	40.74	1.66
	2019	23	10	0.30	21.1	9.12	30.22	1.43
	2020	43	19	0.31	35.7	20.3	55.25	1.55
	2021	44	11	0.20	27	13.1	37.37	1.38
	2022	27	11	0.29	34	20	52.53	1.54

#### 3.2.4 Genetic Diversity

**FIGURE 5:**
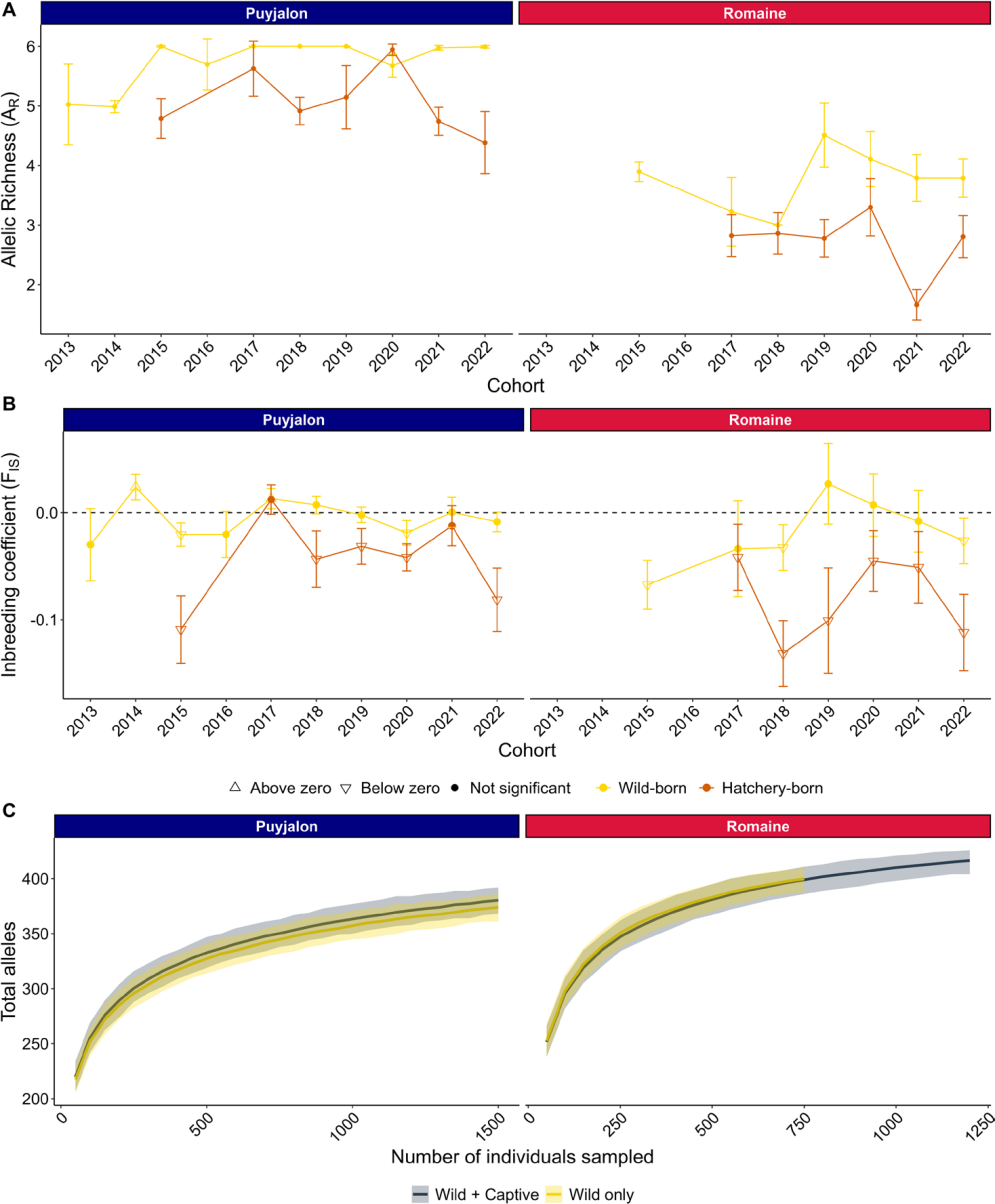
(A) Inbreeding coefficient (*F*_IS_) evaluated for smolt cohorts; (B) allelic richness (*A*_*R*_) evaluated for smolt cohorts. (C) Loess regression of the total number of alleles found by sampling 50 to 1500 (1200 for Romaine River) individuals in wild and in wild + captive bred individuals.

Expected heterozygosity (*H*_*E*_) was significantly lower in Romaine than in Puyjalon (*β* = −0.0241, *p* = 0.008), with an additional reduction observed for hatchery‐origin fish, particularly in Romaine where the interaction term was significant (*p* = 0.021). Observed heterozygosity (*H*_*O*_) showed a similar population effect (*β* = −0.0247, *p* = 0.001) but no significant difference between hatchery and wild fish (Supporting Information S9). Allelic richness (*A*_*R*_) was significantly reduced in Romaine (*β* = −2.115, *p* < 0.001) and in hatchery‐origin individuals (*β* = −0.832, *p* < 0.001, Figure 5A). Inbreeding coefficients (*F*_IS_) were markedly lower in hatchery‐origin individuals (*β* = −0.048, *p* < 0.001), indicating an overall heterozygote excess, and tended to be lower in Romaine than in Puyjalon (*p* = 0.052) (Figure 5B).

Pairwise *F*_ST_ estimates between the Romaine and Puyjalon populations revealed consistent genetic differentiation across cohorts, with values ranging from 0.086 (2020) to 0.125 (2015). The highest differentiation was observed in the earliest cohort (2015; *F*_ST_ = 0.125, 95% CI: 0.108–0.142), followed by a slight decline and stabilization from 2018 to 2021 (*F*_ST_ ≈0.09). A moderate increase was observed again in 2022 (*F*_ST_ = 0.107, 95% CI: 0.095–0.119, Supporting Information S9). Overall, the populations remained genetically distinct across all years.

The addition of captive‐bred individuals contributed to increasing the total number of alleles in the Puyjalon River compared to a scenario in which only wild individuals contributed to reproduction (Figure 5C), but the effect was not significant. For instance, when 1000 offspring were sampled 1000 times in each dataset (with or without captive‐bred individuals), the average number of alleles was 357.34 (CI: 345.00–371.00) for wild individuals alone and 363.28 (CI: 350.00–377.03) when captive‐bred individuals were included. For the Romaine population, the addition of captive‐bred individuals did not affect the total number of alleles (Figure 5C).

### 3.3 Comparison of Survival for Each Incubation Treatment

#### 3.3.1 Egg‐To‐Fry Survival

**FIGURE 6:**
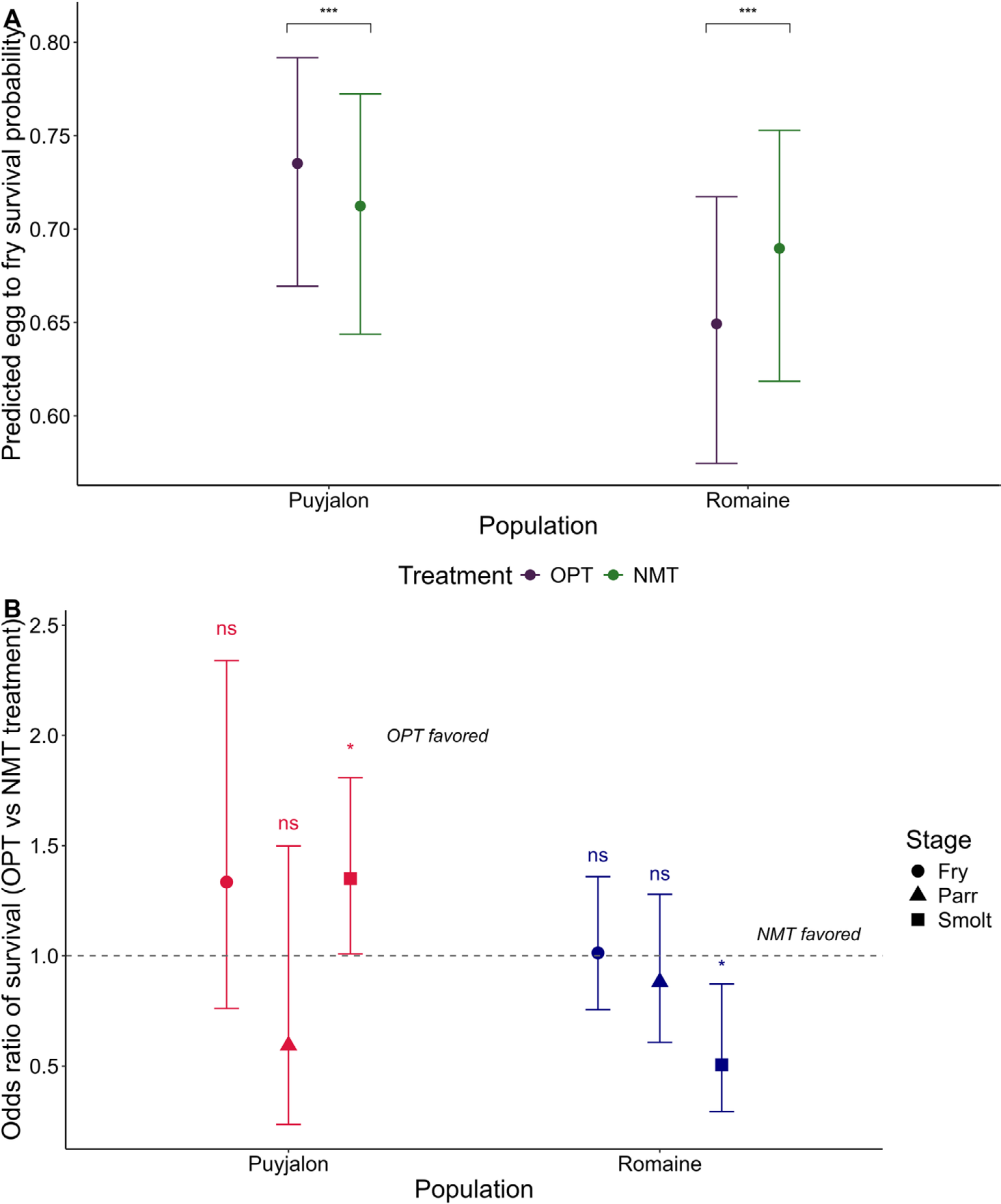
(A) Predicted egg to fry survival probability for each treatment and each population; (B) odds ratio of survival for each stage. Significance indicated with *. Favored treatment is indicated when significant.

The GLMM for predicted egg to fry survival revealed a significant interaction between treatment (OPT or NMT) and origin (*p* < 0.001) (Figure 6A). For Puyjalon families, survival was significant but only slightly higher with the OPT compared to NMT (73.5% vs. 71.2%, *p* < 0.001). The opposite pattern was observed for Romaine families which survived better with NMT than with OPT (69.0% vs. 64.9%, *p* < 0.001). The random effect of year was small but accounted for some variation among years (variance = 0.103, SD = 0.321).

#### 3.3.2 Treatment Survival

A total of 229 fry were assigned to the 2020, 2021, and 2023 stocked families, including 49 individuals from Puyjalon and 180 from Romaine. At this stage, survival did not differ significantly between treatments (LARSA vs. SSRR; OR = 1.33, *p* = 0.313), and no significant treatment × population interaction was detected (OR = 1.32, *p* = 0.394; Figure 6B). Pairwise contrasts confirmed the absence of treatment effects within populations, with no significant differences observed in either Puyjalon (OR = 1.33, *p* = 0.313) or Romaine (OR = 1.01, *p* = 0.929; Figure 6B). Furthermore, 135 parr were assigned to the 2019, 2020, 2022, and 2023 stocked families, including 18 individuals from Puyjalon and 117 from Romaine. No significant effect of treatment was detected (OR = 1.69, *p* = 0.269), and the treatment × population interaction was not significant (OR = 0.67, *p* = 0.438). Consistently, pairwise contrasts revealed no treatment differences within either population (Puyjalon: OR = 0.59, *p* = 0.269; Romaine: OR = 0.88, *p* = 0.507; Figure 6B). Finally, 263 smolts were assigned to 2019 and 2023 stocked families, including 203 individuals from Puyjalon and 60 from Romaine. Both treatment and population significantly influenced survival at the smolt stage, and the treatment × population interaction was significant (OR = 2.67, *p* = 0.0019). Pairwise contrasts revealed opposite treatment effects between populations: survival was higher under LARSA than SSRR in Puyjalon (OR = 1.35, *p* = 0.044), whereas SSRR significantly improved survival in Romaine (OR = 1.98, *p* = 0.014; Figure 6B).

## 4 Discussion

This study evaluates the genetic and demographic outcomes of a long‐term supportive breeding program applied to two genetically distinct Atlantic salmon populations in the Romaine watershed. Broodstock management emerged as a critical determinant of the effective number of breeders and genetic diversity of supplemented individuals. Stocked individuals contributed substantially to smolt cohorts without reducing effective population size, and neither altered population genetic structure nor increased inbreeding. Incubation of eggs in Romaine River water improved survival to the smolt stage, but this benefit was restricted to individuals of Romaine genetic origin and was not detected at earlier life stages. Together, these results provide rare empirical validation of theoretical expectations regarding supportive breeding. By integrating long‐term demographic monitoring, hatchery production data, molecular parentage analyses, and population genetics, this study demonstrates how coordinated cross‐sector conservation efforts can generate robust evidence to guide supplementation strategies while preserving genetic integrity.

### 4.1 Broodstock Capacity to Maintain Genetic Diversity

In this study, the number of breeders (*N*_*b*_) was used as a proxy for the genetic diversity transmitted to stocked individuals because reductions in *N*_*b*_ increase genetic drift and reduce allelic representation (Ferchaud et al. 2016; Sánchez‐Montes et al. 2017). The potential number of breeders (*N*_*b*_pot) was influenced by our capacity to capture smolts in the wild populations. The small number of smolts (54) added to the broodstocks between 2015 and 2018 reflects the difficulty in capturing smolts in those years and therefore, after 2018, parrs were captured directly in the Romaine and Puyjalon River to increase *N*_*b*_pot. To counter the low renewal rate, the use of multi‐year collected and cryopreserved sperm helped maintain a certain amount of genetic diversity despite the reduction of maturing animals. This result is consistent with the recognized value of using cryopreserved sperm in conservation programs (Lozada‐Soto et al. 2026). Broodstock capacity to maintain genetic diversity in supplemented individuals was also primarily constrained by maturation success, which strongly influenced the realized number of breeders (*N*_*b*_real) contributing to each cohort. Due to low maturation, in both populations (53.7% in the Puyjalon and 62.5% in the Romaine), the reduction between *N*_*b*_pot and *N*_*b*_real was 49.6% in the Puyjalon broodstock and 37.0% in the Romaine broodstock, representing the largest decline among the four indices calculated. Hatchery maturation of Atlantic salmon in recovery programs remains poorly documented; however, low maturation rates have been reported and attributed to artificial diets and abiotic rearing conditions such as temperature, salinity, and photoperiod (Fraser 2016; Hansen and Quinn 1998; Mobley et al. 2021; Stark et al. 2014). Consequently, efforts to capture large numbers of broodstock individuals were heavily constrained by hatchery maturation failure, substantially increasing the loss of potential genetic diversity.

Furthermore, our results highlight the importance of balancing family production in stocking programs, as N_b_var was significantly reduced after accounting for offspring variance (−23.6% in Puyjalon and −19.7% in Romaine). Although partial factorial mating can effectively balance family sizes and increase the effective number of breeders while remaining feasible in large hatchery programs (Akers et al. 2023; Busack and Knudsen 2007; Fisch et al. 2015; Fiumera et al. 2004), variation in female age led to unequal reproductive contributions, as egg production strongly depends on size at maturation (Thorpe et al. 1984). In the present program, egg portions were not standardized among females, meaning that larger and older females generally contributed more offspring than smaller females. Consequently, more balanced family sizes could potentially be achieved by predicting female fecundity prior to spawning and adjusting mating allocations accordingly. Given the extensive reproductive data collected during the program, developing population‐specific fecundity models appears feasible and could help reduce variance in family size while maintaining current hatchery capacity. Also, in our system, the number of available males was lower than the number of females, and male reproductive variance was high, especially after 2019. The reduction in *N*_*b*_ was substantially smaller than the decline observed between *N*_*b*_pot and *N*_*b*_real, suggesting that improvements in broodstock maturation represent the greatest opportunity for enhancing genetic outcomes without increasing hatchery workload. Offspring survival from egg to fry further reduced the number of breeders due to highly variable family survival (0.0%–99.2%), although this effect was not significant. Given that egg‐to‐fry survival in natural environments is typically extremely low (2% to 35%) (Dumas and Marty 2006; Mackenzie and Moring 1988; Pauwels and Haines 1994), hatchery incubation likely conserves more genetic variation than egg stocking.

Finally, we evaluated genetic diversity within the broodstock itself. Because the program has been ongoing for 11 years and stocked individuals now represent a substantial proportion of returning fish in both rivers, stocked individuals were incorporated into the broodstock. Although we did not calculate genetic indices separately for stocked individuals, Supplementary analyses (Supporting Information S9) indicate that stocked individuals exhibited slightly lower expected heterozygosity compared to wild‐origin individuals. However, their inclusion in the broodstock did not result in any detectable reduction in overall genetic diversity, as no significant changes in *H*_*E*_, *H*_*O*_, or allelic richness (*A*_*R*_) were detected following their inclusion (Figure 3; Supporting Information S7). This suggests that, despite their slightly reduced individual heterozygosity, stocked individuals did not contribute to a measurable decline in broodstock genetic diversity. Moreover, we observed no increase in inbreeding in the Romaine broodstock over the last 3 years, whereas significant inbreeding was detected in the Puyjalon broodstock in 2020 and 2021. Given the low proportion of stocked individuals in Puyjalon that year (4.2%) and the high turnover in broodstock management (≤ 4 reproductive events per individual), it is unlikely that stocked individuals alone explain the observed inbreeding (Figure 3B) since no inbreeding was detected in 2022–2023 despite a higher proportion of stocked individuals. Allelic richness in the broodstock closely matched that of the wild populations (Puyjalon: *A*_*R*_ *B* = 5.45, *A*_*R*_ *W* = 5.72; Romaine: *A*_*R*_ *B* = 3.97, *A*_*R*_ *W* = 4.20), indicating that broodstocks were representative of their source populations. Nevertheless, comparable genetic diversity at the broodstock and wild levels does not guarantee equivalent diversity in juveniles, as overrepresentation of certain females can reduce offspring diversity (Horreo et al. 2008; Machado‐Schiaffino et al. 2007). Hatchery broodstocks are often founded from a limited number of wild individuals and may therefore exhibit elevated inbreeding that can be transmitted to stocked cohorts if unmanaged (Myers et al. 2001). Inbreeding detected in the Puyjalon broodstock in 2020–2021 followed reduced broodstock renewal between 2015 and 2018. However, partial factorial mating combined with mean kinship minimization and UPGMA‐based avoidance of related crosses prevented inbreeding accumulation in stocked cohorts (Figure 5). Mean kinship remained low across years in both broodstocks, enabling effective genetic management and aligning with outcomes reported in other conservation breeding programs (Hammerly et al. 2016; Montgomery et al. 1997; O'Reilly and Doyle 2007; Putnam and Ivy 2014). Consistent with previous studies, minimizing mean kinship also slowed the loss of genetic diversity relative to random mating (Rabier et al. 2022; Willoughby et al. 2015). Overall, both broodstocks showed comparable genetic diversity to wild populations and their management allowed to ensure effective transmission of this diversity to stocked cohorts in both populations. Nevertheless, this capacity remained highly constrained by incomplete maturation to some extent by uneven reproductive contributions among breeders.

### 4.2 Demographic and Genetic Impact of Stocking

Our results indicate that the Puyjalon smolt population was more abundant than the Romaine population within the Romaine watershed. Interestingly, although not statistically significant, our results suggest a female‐biased sex ratio among Puyjalon smolts, a pattern not observed in fry or parr. This may reflect precocious parr maturation, whereby sexually mature males remain resident in freshwater and do not undergo sea migration, thereby biasing the sex ratio of migrating smolts—a well‐documented phenomenon in Atlantic salmon (Myers 1984; Saunders et al. 1982). Their presence in the Puyjalon population could be linked to the expected higher density of parr compared to the Romaine as documented in other rivers (Baum et al. 2004; Prévost et al. 1992). Precociously mature parr are known to contribute positively to genetic diversity and effective population size (Bouchard et al. 2022; Saura et al. 2008). It could therefore partly explain the higher allelic richness and heterozygosity observed relative to the Romaine population. However, the absence of data on wild adult reproductive success prevents a direct evaluation of this hypothesis.

Stocked individuals were proportionally, but not significantly, more abundant in the Romaine River than in the Puyjalon River (27.1% vs. 20.6%), and this pattern remained stable across years. Given that similar numbers of fry were stocked in both rivers since 2017 and that wild abundance differed markedly (mean smolt abundance: 14,538 in Puyjalon vs. 4065 in Romaine) it is possible that juvenile survival in the Romaine River is lower than in the Puyjalon River. The physical characteristics of the Romaine River are sub‐optimal for Atlantic salmon compared to habitats typically considered optimal for the species (Caron 2018; Mocq et al. 2013). In particular, the Romaine contains extensive sandy reaches (Uanan 2022), which are unsuitable for fry stocking (Finstad et al. 2011; Gibson 1993; Louhi et al. 2008). Moreover, although monitoring of the antenna mat suggests that trapping and stranding may be less important than initially expected, dam operations and large winter water releases can still influence juvenile survival (Halleraker et al. 2003; Hedger et al. 2018; Sauterleute et al. 2016). The salmon‐accessible section of the Romaine River has also been influenced by hydroelectric developments since 2014, resulting in documented modifications of flow and thermal regimes, including reduced spring flood magnitude, increased winter discharge, delayed spring warming, and higher winter water temperatures (Aubé‐Maurice et al. 2025). In particular, the progressive loss of ice cover over several tens of kilometers and temporary increases in algal production associated with successive reservoir impoundments have been identified as additional potential stressors affecting fry survival (Aubé‐Maurice et al. 2025). Hydroelectric developments have also altered sediment dynamics in the river and have been associated with localized sand accumulation in some salmon habitats, potentially affecting habitat quality. By contrast, no comparable hydroelectric developments are present in the Puyjalon River. However, environmental monitoring has been substantially more extensive in the Romaine River than in the Puyjalon River, limiting direct comparisons of habitat conditions and anthropogenic impacts between systems. Finally, nest counts showed no temporal trend, but the similar number of nests in both rivers supports the hypothesis that juvenile survival is lower in the Romaine than in Puyjalon, even for wild individuals.

Our abundance indicators (smolt counts and adult returns) consistently show no increase in population size following juvenile stocking in either river. CMR results indicate stable smolt abundance in Puyjalon across years. The significant decline of smolts in the Romaine largely reflects the high smolt abundance observed in the first 2 years of the program (2013–2014, ~10,000 smolts), whereas since 2015 smolt abundance has remained stable at ~3000. Given the global decline of Atlantic salmon over the last decade—particularly the record‐low adult return rates in 2023–2024 (ICES and WGNAS 2025)—maintaining stable smolt abundance can still represent a relative success. The global review also reports decreased wild smolt production in many Canadian and US rivers, although some systems show increases (ICES & WGNAS). In our study, adult returns remained stable but low (mean ~127), compared with 2001 estimates from GENIVAR (2002) (~300 adults in 1999) but consistent with the wild number of breeders (*N*_*w*_) estimated for 2015–2022 (Table 4). Sea‐age structure was stable through time, indicating that the breeding program did not alter age composition, unlike results reported in the Garonne‐Dordogne program (Fauchet, Bosc, et al. 2026).

In this study, we identified 55 potential offspring assigned to the few sampled adults and did not detect differences in reproductive success (RS) between hatchery‐born adults and wild counterparts. Our results align with a recent study reporting increased fitness in first‐generation descendants of hatchery‐origin Chinook salmon (*Oncorhyncus tshwytscha*) (Dayan et al. 2024). However, the small sample size limits our confidence in those results, especially since lower RS of hatchery‐origin fish is well documented in stocking programs (Bouchard et al. 2022; Christie et al. 2014; Koch et al. 2022; Roth et al. 2025). RS of hatchery fish is of very high importance and is often considered in estimates of Ryman‐Laikre effects (Christie, Marine, French, and Blouin 2012; Hagen and Karlsson 2023) but was considered equal to that of wild fish in this study. Across all analyzed cohorts in both rivers, we found no Ryman–Laikre effect and the *N*_*e*_/*N*_*w*_ ratio ranged from 1.09 to 1.79 in Puyjalon and from 1.34 to 1.66 in Romaine. The absence of this effect reflects good broodstock management before stocking, with not only sufficiently high *N*_*b*_ over many years but also moderate contribution of hatchery‐origin individuals in the populations, which remained lower than levels reported in systems where the Ryman–Laikre effect has been documented. For example, Hagen et al. (2021) identified rivers where hatchery fish exceeded 60% of the population, resulting in a Ryman–Laikre effect despite the *N*_*c*_ of hatchery individuals being similar to the *N*_*c*_ we estimated. Similarly, Christie, Marine, French, and Blouin (2012) and Waples et al. (2016) reported a negative relationship between the proportion of hatchery fish and *N*_*e*_/*N*_*w*_, highlighting the importance of limiting the proportion of stocked fish as achieved in the present program. Importantly, thanks to supplementation efforts, observed total effective size (*N*_*eT*_) values approached or surpassed the conservation benchmark of 50 individuals, historically proposed to limit short‐term inbreeding depression (Franklin 1980). However, subsequent work has suggested that thresholds closer to 100 individuals may be more appropriate for short‐term genetic viability, whereas maintaining long‐term adaptive potential may require effective sizes of several hundred individuals or more (Frankham et al. 2014b, 2014a; Franklin et al. 2014). Despite the absence of a detectable Ryman–Laikre effect, absolute *N*_*e*_ values remained relatively small in both populations and showed no increasing trend, consistent with stable population abundance. Overall, both populations maintained small effective sizes compared to those that have been observed in other rivers in Canada (Ferchaud et al. 2016).

Finally, we showed that genetic diversity (*H*_*O*_, *H*_*E*_, and *A*_*R*_) was lower in the Romaine population than in the Puyjalon population, consistent with the differences in effective size and abundance observed between rivers. Allelic richness was also lower in hatchery‐stocked individuals in both populations (average difference = −0.859), a pattern commonly reported in stocking programs (Almodóvar et al. 2020; Stoeckle et al. 2022). Despite efforts to maintain representativity of the wild populations in both broodstocks, the reduced allelic richness among hatchery‐stocked individuals suggests a mild bottleneck effect (Linløkken et al. 2021). Also, *F*_IS_ values in hatchery‐born individuals indicated significant outbreeding in most cohorts. Outbreeding depression has been a major concern in salmonids when unrelated populations interbreed, often leading to loss of local adaptation (Edmands 2007; Flagg and Nash 1999; Houde et al. 2011; Huff et al. 2011; McClelland and Naish 2007; Rollinson et al. 2014). In our study, population differentiation within the watershed remained stable throughout the program, indicating that the observed outbreeding in hatchery‐stocked individuals is more likely a consequence of breeding design rather than admixture between populations. Indeed, reproduction between closely related individuals was intentionally avoided using mean kinship and UPGMA trees, which can artificially increase outbreeding in the resulting offspring.

### 4.3 Effect of Incubation Treatment on Hatchery Salmon Survival

Population‐specific responses to incubation treatment were observed via differences in survival from the egg to the vesiculated fry stage. While Puyjalon families showed slightly but significantly higher survival under the Optimized Prophylactic Treatment (OPT) (73.3% vs. 72.0% for NMT), Romaine families survived better under the Natural Microbiota Treatment (NMT) (74.0% vs. 67.5% for OPT). For the Romaine River, the results were unexpected as controlled conditions with prophylactic measures are generally assumed to maximize early survival by reducing pathogen exposure and maintaining optimal water quality (Bromage et al. 1992). The superior performance of Romaine families under NMT suggests that these eggs may benefit from early exposure to natural river conditions, potentially through priming of immune responses or reduced stress from handling and environmental manipulation (Saltveit and Brabrand 2013; Smialek et al. 2021). Previous studies have documented population‐specific variation in immune function and stress responses in salmonids linked to local adaptation (Côte et al. 2012; Toews et al. 2019), which could explain why Romaine fish thrived under more natural conditions while Puyjalon fish benefited more from the controlled environment. Alternatively, these differences could stem from maternal effects, family‐specific survival, or egg quality variation between populations (Heath et al. 2003; Rollinson and Hutchings 2010), but these are hard to rule out and our randomized assignment of families to incubation treatments should have minimized such biases.

No significant effect of treatment on survival was detected at the fry or parr stages in either population, suggesting that early incubation environment had limited impact on survival through these intermediate life stages. Sample sizes were relatively small for these stages (226 fry, 125 parr), which may have limited statistical power to detect subtle treatment effects. Interestingly, we observed a significant treatment × population interaction at the smolt stage mirroring the pattern observed for egg‐to‐fry survival. NMT significantly outperformed OPT in Romaine, while OPT led to greater survival in Puyjalon. This convergence of early and late survival patterns suggests that incubation treatment may have persistent effects that become evident at critical life‐history transitions such as smoltification—a physiologically demanding period involving dramatic morphological, behavioral, and physiological changes (Handeland et al. 1996; Morera et al. 2021).

It was expected that Romaine fish incubated in the Romaine River water performed better in the wild than fish incubated at the OPT treatment. Studies on salmonids and other animals have shown that early environmental experience can influence later performance through developmental programming (Jonsson and Jonsson 2014; Lindström 1999; Metcalfe and Monaghan 2001). In the Romaine population, this early “environmental training” under NMT may have better prepared individuals for the challenges of survival in the wild. The fact that Puyjalon individuals performed worse under NMT with Romaine River water suggests the importance of local adaptation in Atlantic salmon and could be explained by the specificity of river microbiota. For example, it has been shown that gut microbiota composition of stocked parrs was principally dependent on the early rearing environment (incubation phase, Lavoie et al. 2021) and that hatchery raised individuals present different gut microbiota than wild individuals (Lavoie et al. 2018). Incubating eggs in the same water as their natal river can therefore help the fish acquire the river's microbiota, thereby facilitating the transition from hatchery to wild environments. It is important to note that the experimental comparison of treatments was conducted over 5 years and therefore does not capture the full range of environmental variability affecting treatment performance. Longer‐term studies would provide more robust assessments of treatment effects across diverse environmental conditions. Also, our study did not assess RS following each treatment, which would be an important next step as a program's success ultimately depends on whether stocked individuals successfully reproduce in the wild (Araki et al. 2007).

## 5 Conclusion

Despite constraints associated with maturation success and unequal reproductive contributions, our results further demonstrate that careful broodstock management can successfully maintain genetic diversity and limit inbreeding both within the hatchery and among supplemented cohorts in the wild. However, although supplementation contributed substantially to smolt production, an increase in Atlantic salmon abundance has not yet been observed in the watershed, which is consistent with broader population declines documented across the species' range. While the program successfully generated valuable biological insights and helped preserve the genetic integrity and demographic contribution of supplemented populations, its potential termination could raise broader questions regarding the long‐term sustainability of restoration outcomes following 11 years of intensive cross‐sector collaboration. The population‐specific responses to incubation treatment highlight the fact that a one‐size‐fits‐all approach to hatchery management can be detrimental to the success of hatchery supplementation programs. The similar or superior performance of minimal prophylactic treatment is particularly encouraging from a cost–benefit perspective, as it requires substantially less infrastructure, energy, and labor than controlled laboratory incubation and therefore may represent a more sustainable and effective approach. In the context of ongoing global salmon declines, the long‐term fate of these populations will ultimately depend on whether the gains achieved through supplementation can be maintained in the absence of continued intervention.

## Funding

The study was conducted with the financial support of Natural Sciences and Engineering Research Council of Canada (NSERCC Grant number: RDCPJ 537349‐18).

## Disclosure

The authors declare that no material from other copyrighted sources has been reproduced in this manuscript.

## Ethics Statement

This work is not under consideration for publication in another journal. The care and use of experimental animals complied with Canada and Université Laval animal welfare laws, guidelines, and policies as approved by the Comité de Protection des Animaux de l'Université Laval (CPAUL) (permit reference number: 2021‐783). Also, individuals transferred at the Laboratoire Aquatique de Recherche en Sciences Médicales et Environnementales were euthanized after four reproductions, as approved by the CPAUL.

## Conflicts of Interest

The authors declare no conflicts of interest.

## Supporting information


**Supporting Information: S1.** Table with all 52 microsatellite loci used for genetic characterization from the panel of 101 loci (Bradbury et al. 2018)—loci marked with an asterisk were removed from analyses.
**Supporting Information:** S2. (A) Number of loci on each chromosome; (B) number of alleles per locus; (C) FST per locus.
**Supporting Information:** S3. Mean Kinship for all individuals that reproduced in 2023 at the LARSEM. Genotypes were analyzed using the R packages adegenet (Jombart, 2008) and related (Pew et al. 2015) to compute pairwise relatedness coefficients. Mean kinship values were derived for each individual by averaging pairwise relatedness across all potential mates within the population.
**Supporting Information:** S4. UPGMA tree for all Romaine individuals that reproduced in 2023 at the LARSEM. The proportion of shared alleles was also calculated and used to generate UPGMA trees for each population with the phangorn package (Schliep, 2010). These trees provided visual representations of genetic relationships and combined with mean kinship values guided breeder selection to ensure minimal relatedness among mating pairs.
**Supporting Information:** S5. Number of individual (male or female and adult or parr or smolt) that joined the broodstock between 2013 and 2022 for each population.
**Supporting Information:** S6. (A) Number of offspring N, number of offspring per male (Km) and female (Kf) for each reproduction year. (B) Density curve in number of offspring for male and female from 2014 to 2023. (C) Variance in number of offspring in male (Vm) and female (Vf) for each reproduction year. (D) Survival rate from egg to fry for each year of reproduction.
**Supporting Information:** S7. (A) Number of breeders originating from the wild or the hatchery between 2014 and 2023. (B) Expected heterozygosity for each reproduction year in each population. (C) Observed heterozygosity for each reproduction year in each population and each population.
**Supporting Information:** S8. Table with number of wild (Wild) and hatchery (Hatchery) smolts genotyped, proportion of hatchery smolts (x), the effective number of wild smolts (Nw) as well as lower and upper confidence intervals at 95%, the effective number of hatchery smolt calculated with LD methods and 95% CI, the effective number of hatchery smolts calculated with the pedigree methods (Nc [ped]), the total effective population size as derived from (4) (Ne [ped]), a measurement of whether stocking caused a decrease in the total effective population sizes (Ne [ped]/Nw).
**Supporting Information:** S9. (A) Expected heterozygosity (HE) evaluated for smolt cohorts; (B) observed heterozygosity (HO) evaluated for smolt cohorts. (C) pairwise Nei's FST calculated for smolt cohorts.

## Data Availability

Data for this study are hosted in the following GitHub repository: https://github.com/louarnfauchet‐hub/salmosalar_Romaine_stocking.git.
