# Supplementary material for: Multidisciplinary Evaluation of a 10‐Year Restoration Program for Two Endangered Atlantic Salmon ( Salmo salar ) Populations
Source: Evol Appl. 2026 Jun 29;19(7):e70289. doi: 10.1111/eva.70289 (PMC13312309; doi:10.1111/eva.70289)
Supplement: Supplementary file 1 — Supporting Information: S1. Table with all 52 microsatellite loci used for genetic characterization from the panel of 101 loci (Bradbury et al. 2018)—loci marked with an asterisk were removed from analyses. Supporting Information: S2. (A) Number of loci on each chromosome; (B) number of alleles per locus; (C) FST per locus. Supporting Information: S3. Mean Kinship for all individuals that reproduced in 2023 at the LARSEM. Genotypes were analyzed using the R packages adegenet (Jombart, 2008) and related (Pew et al. 2015) to compute pairwise relatedness coefficients. Mean kinship values were derived for each individual by averaging pairwise relatedness across all potential mates within the population. Supporting Information: S4. UPGMA tree for all Romaine individuals that reproduced in 2023 at the LARSEM. The proportion of shared alleles was also calculated and used to generate UPGMA trees for each population with the phangorn package (Schliep, 2010). These trees provided visual representations of genetic relationships and combined with mean kinship values guided breeder selection to ensure minimal relatedness among mating pairs. Supporting Information: S5. Number of individual (male or female and adult or parr or smolt) that joined the broodstock between 2013 and 2022 for each population. Supporting Information: S6. (A) Number of offspring N, number of offspring per male (Km) and female (Kf) for each reproduction year. (B) Density curve in number of offspring for male and female from 2014 to 2023. (C) Variance in number of offspring in male (Vm) and female (Vf) for each reproduction year. (D) Survival rate from egg to fry for each year of reproduction. Supporting Information: S7. (A) Number of breeders originating from the wild or the hatchery between 2014 and 2023. (B) Expected heterozygosity for each reproduction year in each population. (C) Observed heterozygosity for each reproduction year in each population and each population. Supporting Information: S8. Table [file EVA-19-e70289-s001.docx]

**Supplementary materials**

**S1:** Loci used for genetic characterization from the panel of 101 loci (Bradbury et al. 2018) – loci marked with an asterisk were removed from analyses

| **Locus** | **Multiplexe** | **Amorce sens** | **Amorce anti-sens** | **Motif répété** | **Amplicon (pb)** | **Chromosome** |
| --- | --- | --- | --- | --- | --- | --- |
| **NGS-SsaD486** | 1a | TGCAGTCCAATAATATCCCCGT | CCCTGCATGACTCGGATAAC | AGAT | 126 | Inconnu |
| **NGS-SSsp2210** | 1a | CACATTCACTGCAAAATAAAGCT | TGGGATTCAATAAAGGTAAGTAAGT | AGTT | 100 | Inconnu |
| **Ssa-1.5*** | 1a | GCGTTATGTGCTTGCATGC | ACCACCGTACTCAGCTTATCC | ATT | 110 | 1 |
| **Ssa-1.7** | 1a | AGAACACAACAGAACCAGGTAC | CTCGAACACACTTCCAACCC | GAT | 119 | 1 |
| **Ssa-1.8*** | 1a | AGGCCAAAGAAATCCTGCAC | ACTGACCCAAACACGCAAATAG | ATC | 120 | 1 |
| **Ssa-2.1** | 1a | AGACTCCACCTGCCTTGTTC | CTCACTGTCAGAGCATGCG | ATT | 91 | 2 |
| **Ssa-2.2** | 1a | TGGCCATTCTCCAGAGCTAG | CCACCAAAGGAGAGTACGTG | CTT | 94 | 2 |
| **Ssa-2.7** | 1a | CCCAGACTTCCCACTCTCTATG | GGACACAGAACCTTGAACGG | CTG | 119 | 2 |
| **Ssa-5.2** | 1a | AACTTGCGTGATGATGTGGC | GCTGGCCATGTTCTTCTGTG | CTT | 92 | 5 |
| **Ssa-6.2** | 1a | GGAGAAGAGGAGATGGAACTTG | ACACCTGACAATACCACACC | TGA | 98 | 6 |
| **Ssa-7.1*** | 1a | CCACTCCCACGAATGATGTTC | GGAGGCCACATTGCAGTC | AAC | 98 | 7 |
| **Ssa-9.3** | 1a | GCCAACCACCGTTAAACCTC | TCAGCAGTTCCCAATATTTCCC | AAG | 92 | 9 |
| **Ssa-9.8** | 1a | GCGTCGACTGCCATTCAAC | TGTCCTTGCTTTCTCCGTGG | AAG | 118 | 9 |
| **Ssa-10.1** | 1a | GGTCCTCCAGTACCTCCAAC | AATCTGGTGAGTTCGTCCGG | GTT | 83 | 10 |
| **Ssa-10.4*** | 1a | GGTGAAATGTAGCCTGCATG | ACACACTGCTATATGTGTGG | AAT | 121 | 10 |
| **Ssa-11.2** | 1a | AAAGTTTGTTTGTGGACCGC | CGGACAGTTTCTTGGACTTC | AAG | 115 | 11 |
| **Ssa-11.3** | 1a | AGCGTGTGTGTCGTTCAATAC | ATGTTTCACCTCTGCGTCAC | AAC | 118 | 11 |
| **Ssa-11.5** | 1a | GTGTGCCGTTCTATCGCTG | CCTAAAGAAATGCCAGAGTCCG | GAT | 136 | 11 |
| **Ssa-11.6*** | 1a | TTAACCTGCTCTACCTCTCG | ACATCACCACACCTATCTTC | TGA | 136 | 11 |
| **Ssa-12.5** | 1a | TCTCCTTCCTCGATCAGCTC | AATGTGTCGCCTTCCCACC | ATC | 140 | 12 |
| **Ssa-14.2** | 1a | GGGCATGATCTCGACACC | AGGAATGAGTAAGCTGGCTAAG | ATC | 99 | 14 |
| **Ssa-14.6** | 1a | AGTCAAGAAAGTCACTGCCC | GGAATGGCAAACAGAAAGGG | ATT | 128 | 14 |
| **Ssa-15.1** | 1a | TTTCTTTGTGTGTTGTGCCC | CAGCTGTGGTTCCTCTGGG | CCT | 96 | 15 |
| **Ssa-15.3** | 1a | GCTAACGAATGACAGCTTGC | CATTAGTAAGACTGGCAGCAG | TTG | 102 | 15 |
| **Ssa-15.7** | 1a | GATGTGATGGCAGTGCTATG | CAGCAACAAGGTCAATCTCC | TGA | 120 | 15 |
| **Ssa-19.1** | 1a | TGTGCAAACGCCATGATACC | CCATGACAGCTCCATCCGG | GTA | 91 | 19 |
| **Ssa-19.2** | 1a | GTGACCCAAAGTGCTGCTG | CTCCAGACACCAGCACCTC | GCT | 95 | 19 |
| **Ssa-19.3** | 1a | ACGTCCTGACAGTTATCCTTG | GTCTTGTCATGGCTGTGCTC | TTC | 97 | 19 |
| **Ssa-20.2** | 1a | TCTTCCCTCTTCTGCAGCAG | AGCTCTGGACACCACACTG | GTG | 97 | 20 |
| **Ssa-23.2*** | 1a | GGTGGTTGTTTCTAGTGAGGG | GCACCTCTAAAGCACCATGG | CTT | 101 | 23 |
| **Ssa-25.2** | 1a | TGCAGGAAGACTCTGAAAGG | AGGTGGGTGTTGTACATCAG | GAT | 124 | 25 |
| **Ssa-4.d44** | 2a | TTGGGTCTTAATGGCACCTG | GCTTTGGTTCCCTGAGAGTG | AC | 82 | 4 |
| **Ssa-5.6*** | 2a | GTGCAGCTGTTCCTCACTTC | GGGACAGGCGTAGAAATCG | TAT | 134 | 5 |
| **Ssa-6.7*** | 2a | GCAAATCAGCATTCAGGGC | CAGCTGATCGAACTGAATGGG | TAA | 132 | 6 |
| **Ssa-7.12** | 2a | CACTCCCTGACACGTTAACAC | CACTTCCTGACAAACATGCAC | ACT | 99 | 7 |
| **Ssa-9.13** | 2a | ATCCACACCTCTCTTGCCAC | GATCACCATCGTTACCATCCC | AGG | 109 | 9 |
| **Ssa-10.2** | 2a | TGATCCTCTTCACCACCCTG | CTGAAGACTCCTCCCTCACC | AAT | 99 | 10 |
| **Ssa-11.8** | 2a | AAAGGACCCAGAACGTACAG | ACCACACAGTACCCTCAATG | ATA | 146 | 11 |
| **Ssa-13.8** | 2a | TGACGAGACAAGATTCAGGTTG | GACCTATGCAACCACCAACG | GTT | 138 | 13 |
| **Ssa-14.3** | 2a | TCAACCTAAACCCTCTGCCC | AATCATCACATTCCACAGCAAC | AAT | 108 | 14 |
| **Ssa-14.5** | 2a | CCAGGAGGCCTTCACATG | CCTCCTGGCAATGCTGTATAG | AAT | 119 | 14 |
| **Ssa-14.8** | 2a | AAACATTGATTTGGCTCTGTC | TATTGCACCATCCCGTTCTC | TAT | 154 | 14 |
| **Ssa-17.1** | 2a | CATCTTCCGGTTCGCTCAAC | GTCATGACCTGTGCAACCAG | ATT | 99 | 17 |
| **Ssa-18.7** | 2a | TGCAGGTTGTGGTCATGTTG | CACATTCTGTCCATTCGGCC | TTA | 158 | 18 |
| **Ssa-20.d56*** | 2a | GAGGTCAAGGTTTCCACTGG | TAGCTGCTCTCTGTTCTGGG | AG | 76 | 20 |
| **Ssa-21.2** | 2a | CTGTCCAAATTGCAGGCTTG | GCCTAATTTGCCTACTCCTGTC | TAT | 120 | 21 |
| **Ssa-22.2*** | 2a | AGTGGTTGCTTTGGTTCTCC | GGATAAAGCGGACCAAGACG | AAT | 117 | 22 |
| **Ssa-22.5** | 2a | GTGACGTCTGGAATTGTGAC | GATCCAATCAACACCGGTAG | AAT | 138 | 22 |
| **Ssa-22.9** | 2a | CAAATGCCACACGACCTGAC | GGTCAACCGCTCTGCATATAG | ATT | 112 | 22 |
| **Ssa-22.d31** | 2a | AGTTTAGTAGGGCCTGCGTG | ACATTCTTCTGTCACAGCCTG | GT | 75 | 22 |
| **Ssa-25.11** | 2a | GGGTCCATGAGAAAGGCAAC | TGGGATCCACACCTGACAAC | ATT | 119 | 25 |
| **Ssa-26.1** | 2a | TCACGCATAACCTTAGACAACC | AATGCCAACCCTGTTACAGC | ATT | 117 | 26 |

**S2**: A: Number of loci on each chromosome; B: Number of alleles per locus; C: F_ST_ per locus

**
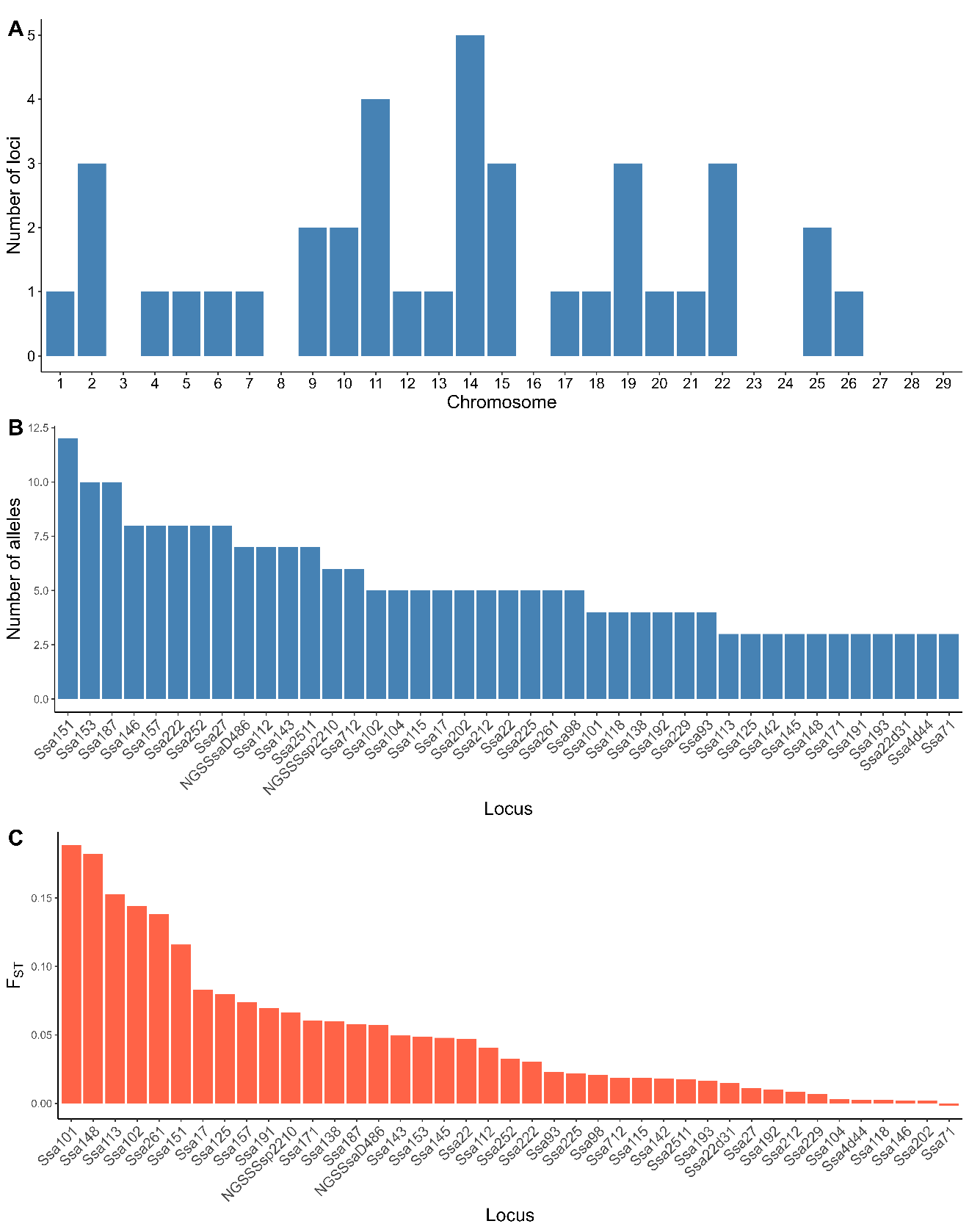
**

**S3**: Mean Kinship for all individuals that reproduced in 2023 at the LARSEM. Genotypes were analyzed using the R packages adegenet (Jombart, 2008) and related (Pew et al., 2015) to compute pairwise relatedness coefficients. Mean kinship values were derived for each individual by averaging pairwise relatedness across all potential mates within the population.


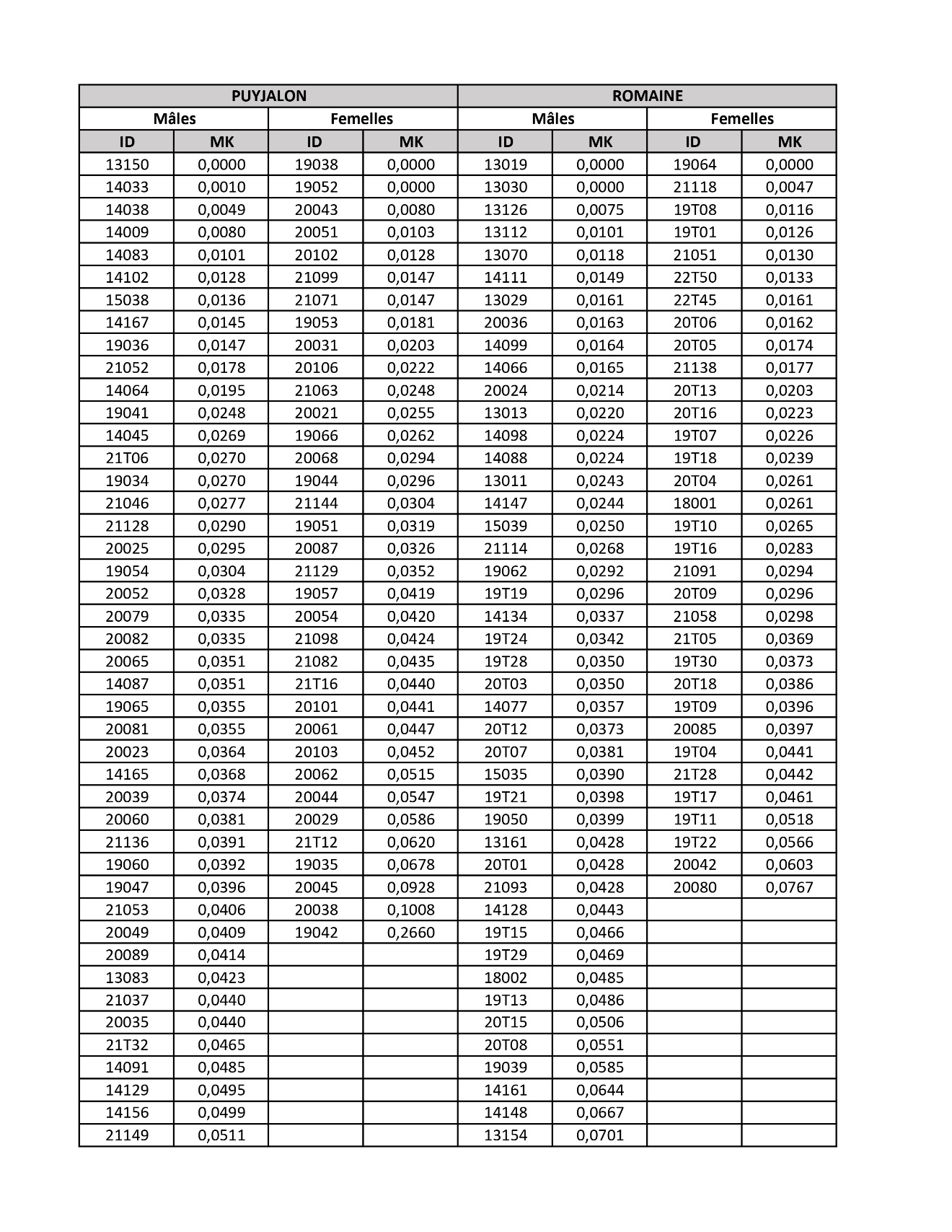


**S4**: UPGMA tree for all Romaine individuals that reproduced in 2023 at the LARSEM. The proportion of shared alleles was also calculated and used to generate UPGMA trees for each population with the phangorn package (Schliep, 2011). These trees provided visual representations of genetic relationships and combined with mean kinship values guided breeder selection to ensure minimal relatedness among mating pairs.


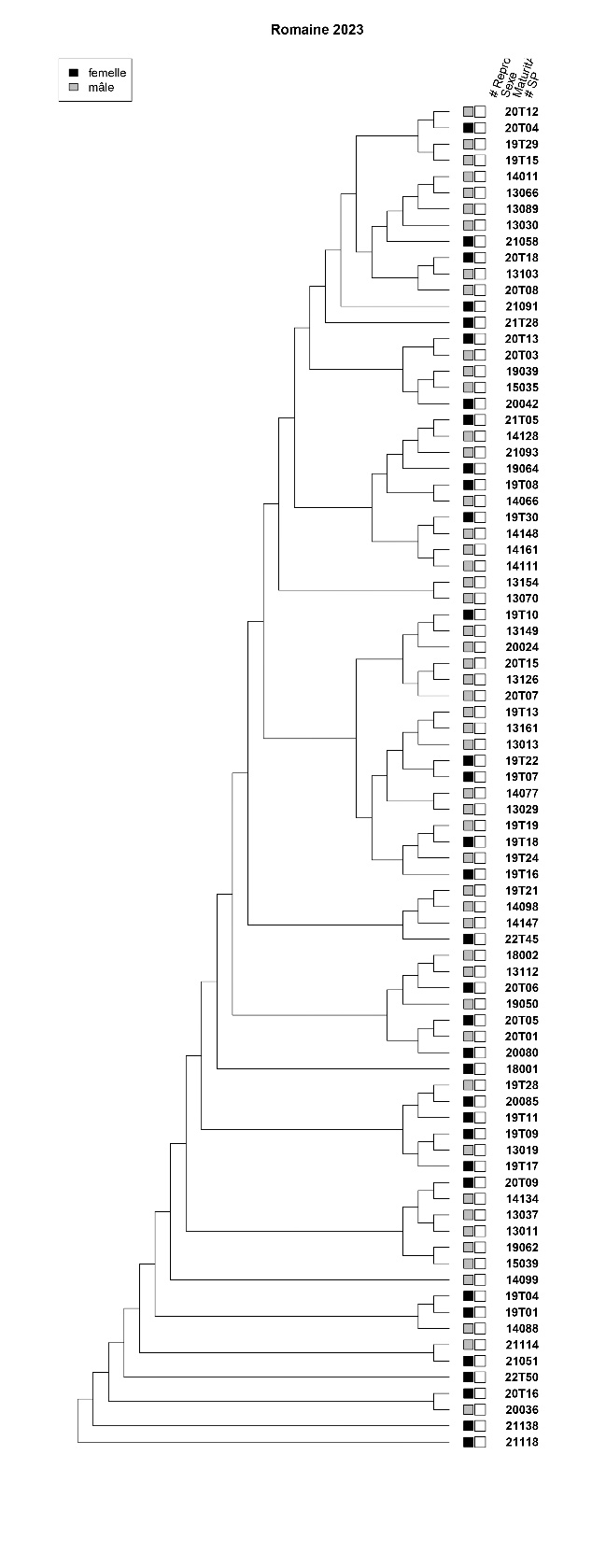


**S5**: Number of individual (male or female and adult or parr or smolt) that joined the broodstock between 2013 and 2022 for each population.


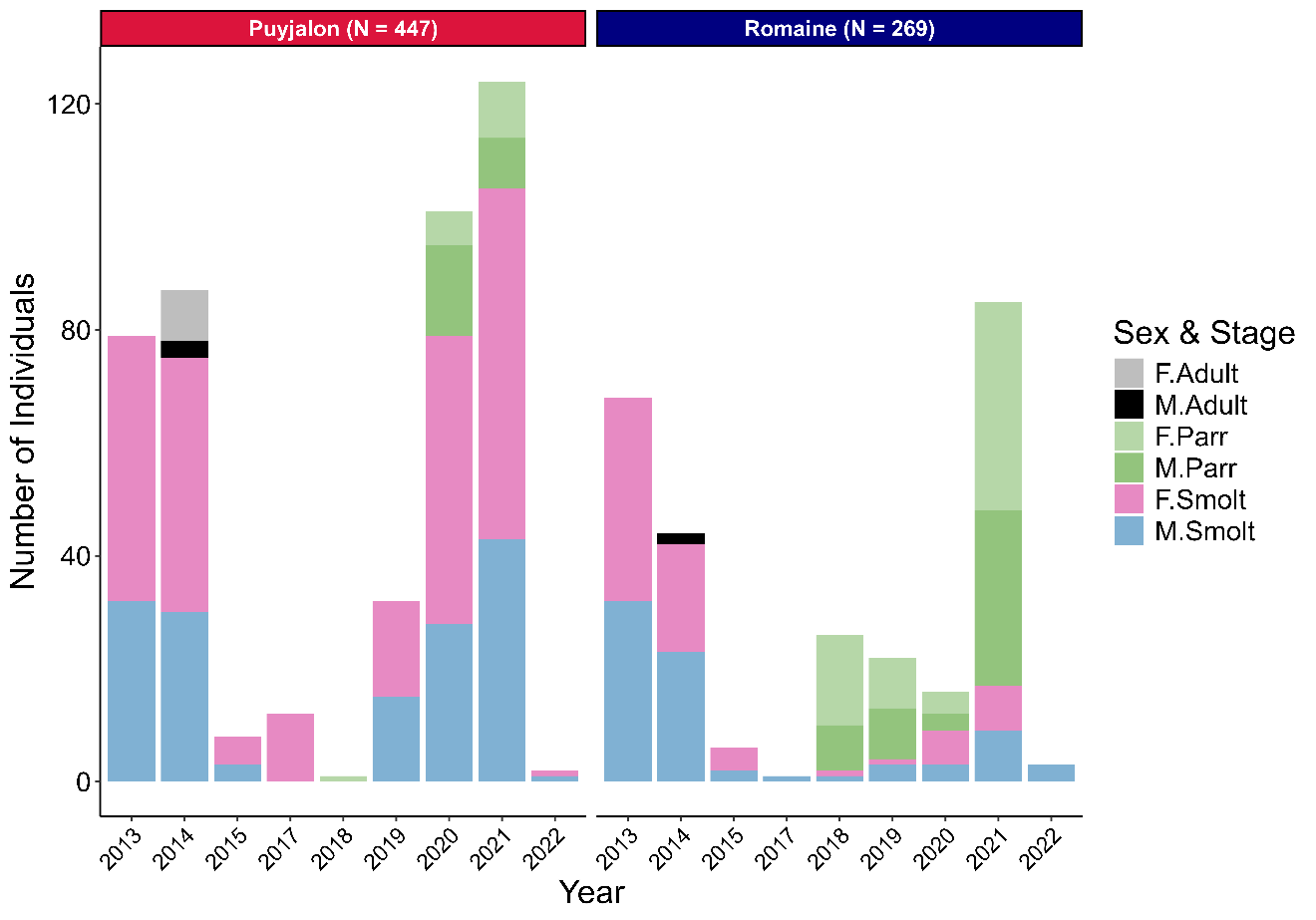


**S6**: A: Number of offspring N, number of offspring per male (Km) and female (Kf) for each reproduction year. B: Density curve in number of offspring for male and female from 2014 to 2023. C: Variance in number of offspring in male (Vm) and female (Vf) for each reproduction year. D: Survival rate from egg to fry for each year of reproduction.


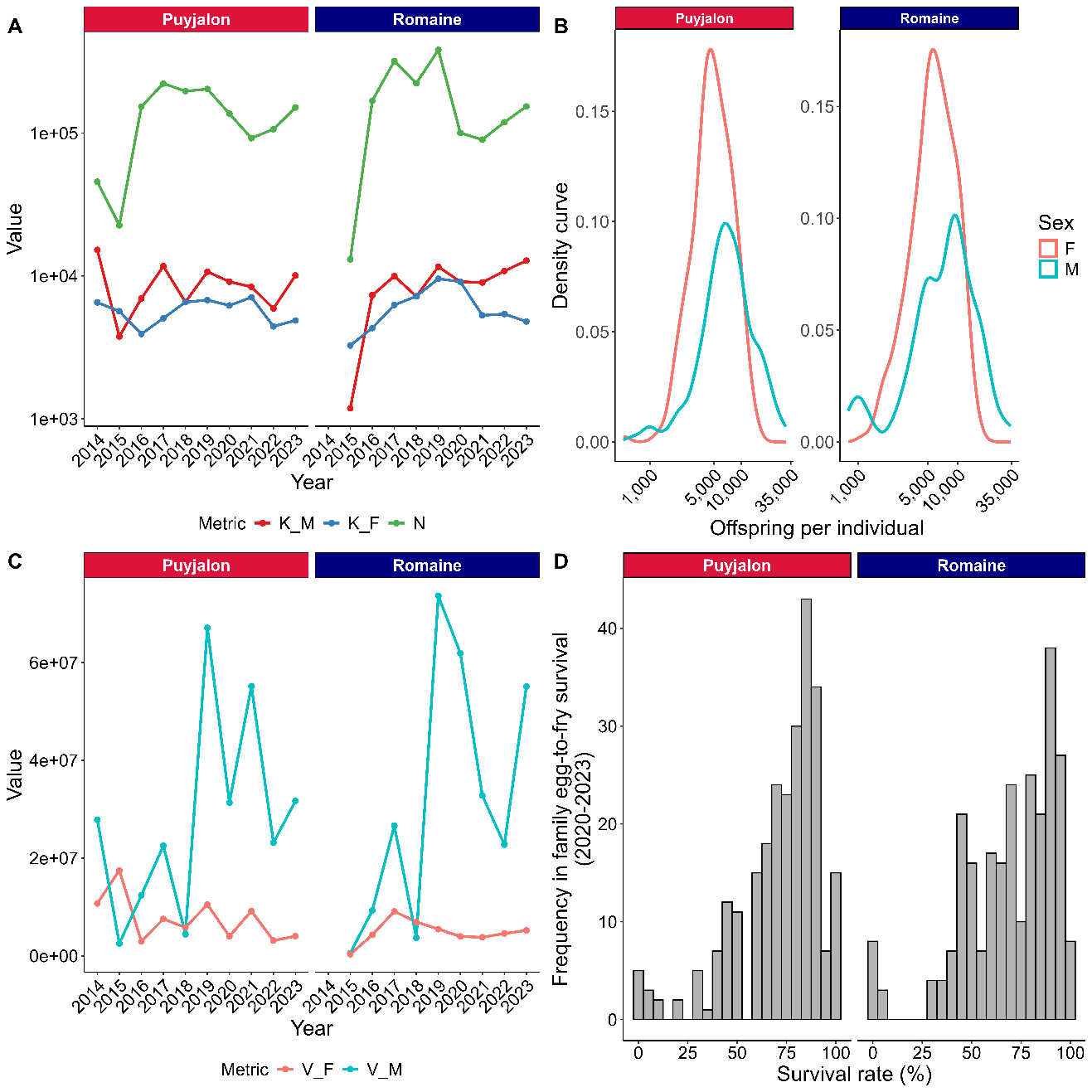


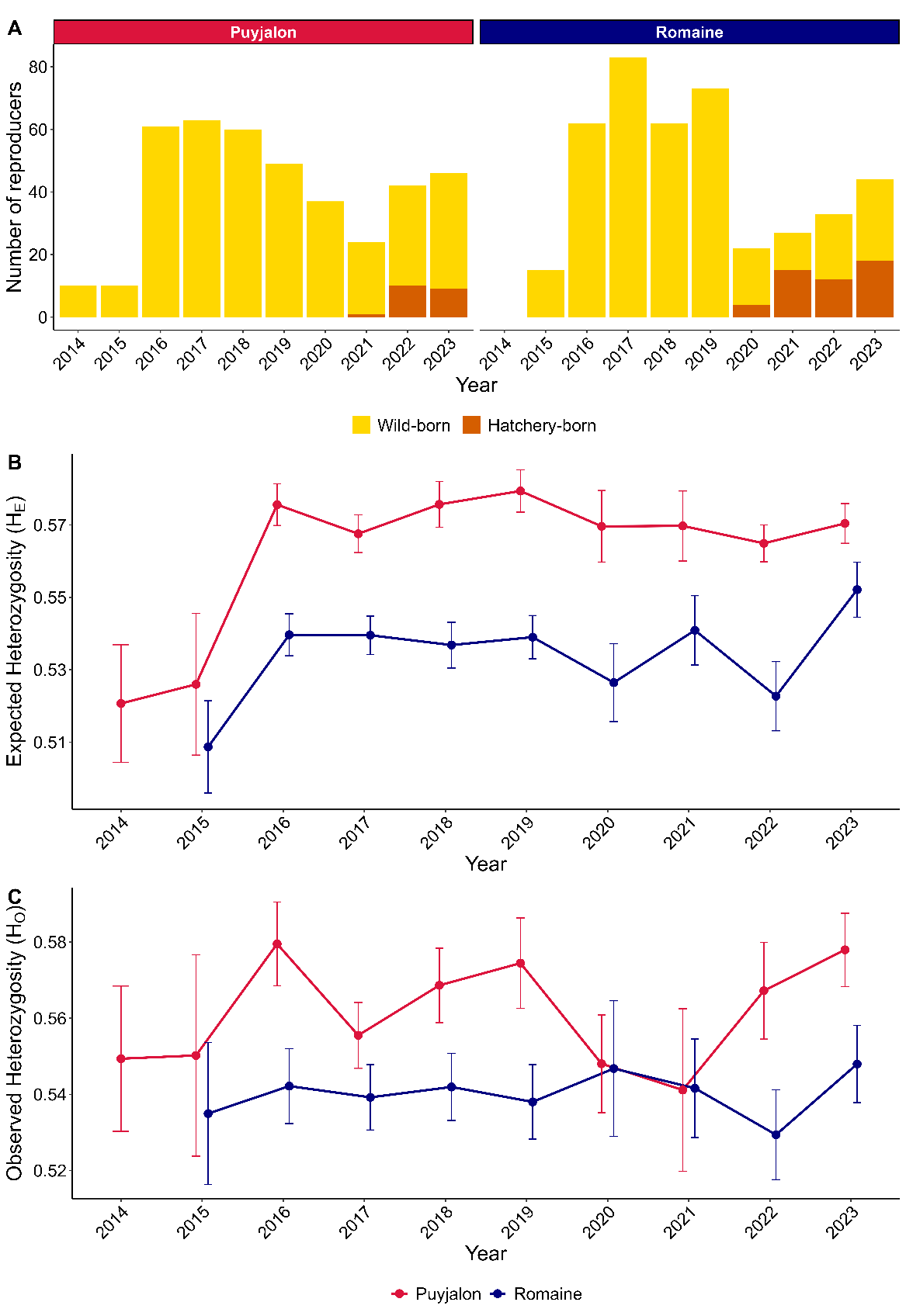
**S7**: A: Number of breeders originating from the wild or the hatchery between 2014 and 2023. B: Expected heterozygosity for each reproduction year in each population. C: Observed heterozygosity for each reproduction year in each population and each population.

**S8:** Number of wild (Wild) and hatchery (Hatchery) smolts genotyped, proportion of hatchery smolts (x), the effective number of wild smolts (N_w_) as well as lower and upper confidence intervals at 95%, the effective number of hatchery smolt calculated with LD methods and 95% CI, the effective number of hatchery smolts calculated with the pedigree methods (N_c_ (ped)), the total effective population size as derived from (4) (N_e_ (ped)), a measurement of whether stocking caused a decrease in the total effective population sizes (N_e_ (ped)/Nw)

| Population | Cohort | Wild | Hatchery | x | Nw | Nw (Lower) | Nw (Upper) | Nc (LD) | Nc (LD Lower) | Nc (LD Upper) | Nc (ped) | Ne (ped) | Ne(ped)/Nw |
| --- | --- | --- | --- | --- | --- | --- | --- | --- | --- | --- | --- | --- | --- |
| Puyjalon | 2015 | 94 | 18 | 0,16 | 44,7 | 40,8 | 49 | 10,8 | 8,9 | 13,3 | 5,91 | 49,68 | 1,11 |
|  | 2016 | 36 | 3 | 0,08 | 43,4 | 36 | 53,6 | NA | NA | NA | 3,73 | 47,13 | 1,09 |
|  | 2017 | 189 | 104 | 0,35 | 75,4 | 69,8 | 81,5 | 34,7 | 32,2 | 37,6 | 41,9 | 117,30 | 1,56 |
|  | 2018 | 217 | 26 | 0,11 | 69 | 64,4 | 73,9 | 32,3 | 25,9 | 41,8 | 29 | 83,67 | 1,21 |
|  | 2019 | 215 | 48 | 0,18 | 52,6 | 49,3 | 56,2 | 24,1 | 21,6 | 27,1 | 31,4 | 72,64 | 1,38 |
|  | 2020 | 145 | 85 | 0,37 | 24,2 | 22,7 | 25,8 | 21,9 | 20,3 | 23,6 | 21,4 | 43,85 | 1,81 |
|  | 2021 | 108 | 41 | 0,28 | 37,5 | 34,7 | 40,7 | 13,2 | 11,8 | 14,7 | 19,6 | 55,95 | 1,49 |
|  | 2022 | 112 | 29 | 0,21 | 46,2 | 42,5 | 50,3 | 10,7 | 9,5 | 12,1 | 11,7 | 57,90 | 1,25 |
| Romaine | 2018 | 27 | 10 | 0,27 | 24,6 | 20,9 | 29,3 | 17 | 11,8 | 27,4 | 25,2 | 40,74 | 1,66 |
|  | 2019 | 23 | 10 | 0,30 | 21,1 | 17,9 | 25,1 | NA | NA | NA | 9,12 | 30,22 | 1,43 |
|  | 2020 | 43 | 19 | 0,31 | 35,7 | 30,9 | 41,9 | 12,6 | 10,7 | 14,9 | 20,3 | 55,25 | 1,55 |
|  | 2021 | 44 | 11 | 0,20 | 27 | 24 | 30,5 | NA | NA | NA | 13,1 | 37,37 | 1,38 |
|  | 2022 | 27 | 11 | 0,29 | 34 | 28,3 | 41,9 | 15,2 | 11 | 22,4 | 20 | 52,53 | 1,54 |


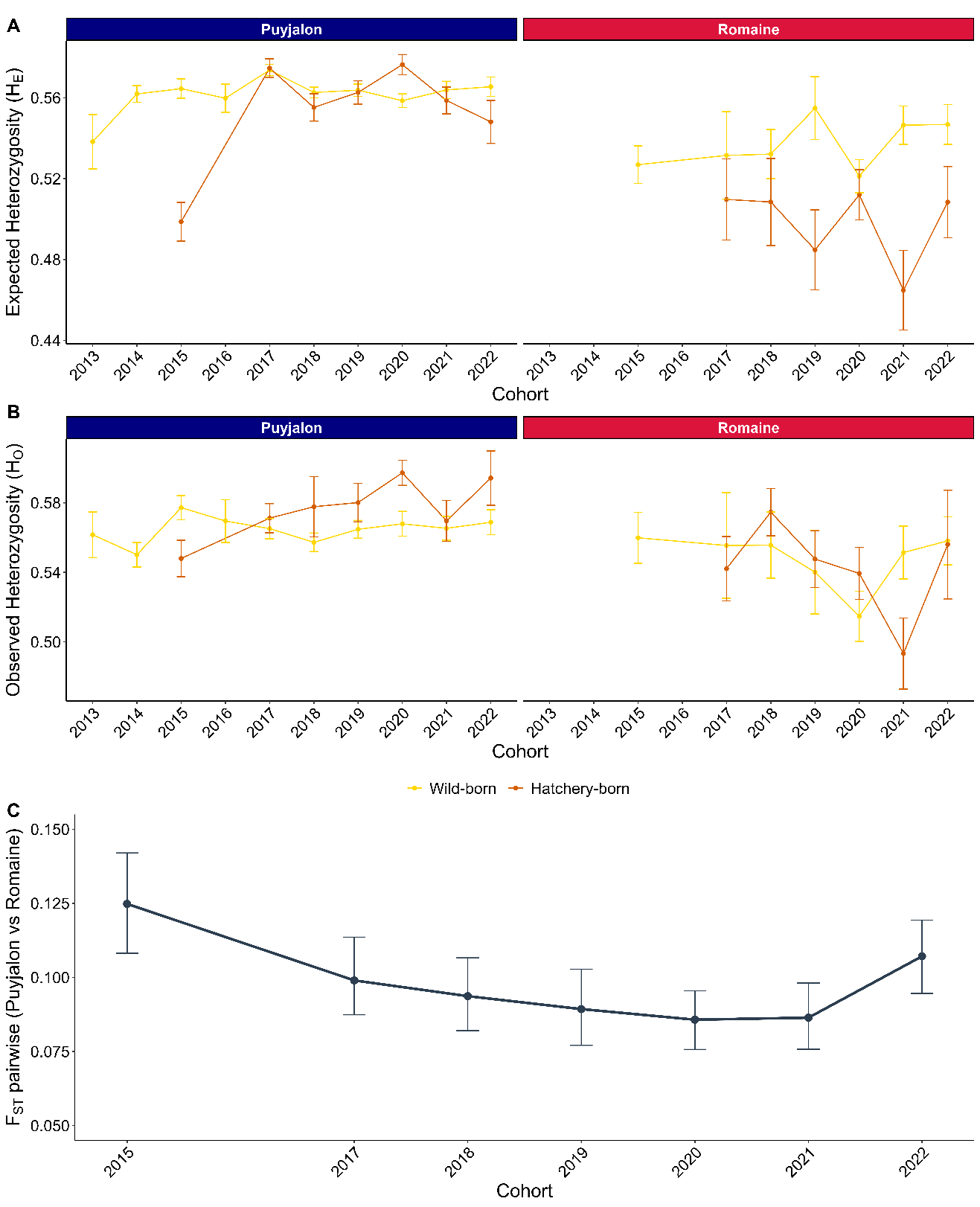
**S9:** A: Expected heterozygosity (H_E_) evaluated for smolt cohorts B: Observed heterozygosity (H_O_) evaluated for smolt cohorts. C: Pairwise Nei’s F_ST_ calculated for smolt cohorts.
